# Raw Milk as a Source of *Campylobacter* Infection: Isolation and Molecular Identification of *Campylobacter coli* and *Campylobacter jejuni* in Ecuador

**DOI:** 10.3390/pathogens14111155

**Published:** 2025-11-13

**Authors:** Andrea Padilla-Cerda, Anthony Loor-Giler, Byron Puga-Torres, Silvana Santander-Parra, Luis Núñez

**Affiliations:** 1 Facultad de Ingeniería y Ciencias Aplicadas, Carrera de Ingeniería en Biotecnología, Universidad de Las Américas (UDLA), Antigua Vía a Nayón S/N, Quito EC 170124, Ecuador; andrea.padilla.cerda@outlook.com; 2 Laboratorios de Investigación, Dirección General de Investigación, Universidad de las Américas (UDLA), Antigua Vía a Nayón S/N, Quito EC 170124, Ecuador; a.abel.loor.giler@gmail.com; 3 Facultad de Ciencias Veterinarias, Universidad de Buenos Aires, Av. Chorroarín 280, Buenos Aires 1427, Argentina; 4 Facultad de Medicina Veterinaria y Zootecnia, Universidad Central del Ecuador, Jerónimo Leyton s/n y Gilberto Gatto Sobral, Quito EC 170521, Ecuador; bpuga@uce.edu.ec; 5 Facultad de Ciencias de la Salud, Carrera de Medicina Veterinaria, Universidad de Las Américas, Antigua Vía a Nayón S/N, Quito EC 170124, Ecuador; silvanahsp@yahoo.com; 6 One Health Research Group, Facultad de Ciencias de la Salud, Universidad de Las Américas, Quito EC 170124, Ecuador

**Keywords:** foodborne gastroenteritis, *Campylobacter* spp., *C. coli*, *C. jejuni*, raw milk, PCR

## Abstract

The consumption of raw milk has been demonstrated to carry a potential risk of transmission of *Campylobacter* spp., with *Campylobacter jejuni (C. jejuni)* and *Campylobacter coli (C. coli)* being the major causes for foodborne gastroenteritis cases. The present study assessed the prevalence and species distribution of *Campylobacter* spp. in 633 raw milk samples collected over a one-year climatic cycle from small, medium, and large producers in Pichincha and Manabí, Ecuador. Samples were augmented and analyzed by qPCR for *Campylobacter* spp., while species identification was performed by duplex PCR and confirmed by 16S rDNA sequencing. The average prevalence of *Campylobacter* spp. was 49.9% (316/633), with a higher detection rate in Manabí (57.6%, 182/316) compared to Pichincha (42.4%, 134/316). *C. coli* was the most prevalent species, accounting for 46.2% (146/316) of the cases, followed by *C. jejuni* at 23.1% (73/316), co-contaminations at 13.3% (42/316), and non-identified *Campylobacter* at 44.0% (139/316). Phylogenetic analysis was employed to confirm species identity, thereby confirming the presence of *Campylobacter fetus* and *Campylobacter lari*. The increased diversity and frequency of isolates in Manabí, particularly during periods of elevated temperature, imply that coastal environmental conditions and production practices promote the persistence of bacteria. The findings of this study indicate a high prevalence of *Campylobacter* in Ecuadorian raw milk, posing a significant health risk to the population and underscoring the need for enhanced hygiene practices and continuous monitoring to mitigate public health risks.

## 1. Introduction

In Ecuador, the dairy industry is an important economic activity, which produces 5.3 million liters of raw milk per day [1]. Most milk production is concentrated in the Sierra region, accounting for 79.5% of the total, followed by the Coast (16.4%) and Amazon (4.1%) regions [2]. Pichincha, Azuay, and Manabí are the provinces with the highest milk production, with Pichincha exceeding one million liters produced per day in 2021 [3]. This industry, directly or not, involves 10% of the Ecuadorian population, with a total of 285,000 producers, 80% of which are small farms, and 20% are medium and large farms [4]. Raw milk, defined as milk that has not undergone any thermal process—such as pasteurization—to ensure its safety, has a high content of macro- and micronutrients. These nutrients, in combination with their neutral pH, create an optimal environment for the proliferation of a diverse array of microorganisms, including some zoonotic pathogens [5,6]. The primary cause of contamination in raw milk is improper milking practices, particularly the presence of fecal contaminants in the udder [7]. It should be noted that even milk from healthy cows may contain pathogenic microorganisms, posing a potential risk to consumer health [5].

Despite the global implementation of pasteurization systems in the early 21st century, raw milk consumption remains common in certain niches, with an estimated prevalence of 1 in 100 people worldwide [8]. Its consumption is attributed to cultural practices or common assumptions that it is healthier and easier to digest [9]. In 2013, in the United States, raw milk consumption was reported to account for 2.1% of the causes of foodborne infectious diseases, revealing that it remains a public health problem [10]. The most prevalent pathogens identified in raw milk samples include various species of *Cryptosporidium*, *Listeria monocytogenes*, Shiga toxin-producing *Escherichia coli* (STEC), *Salmonella* enterica, and *Campylobacter* species [11]. Within this group, *Campylobacter* spp. deserves special consideration due to its high prevalence. The presence of the contaminant has been detected in approximately 25% of raw milk and dairy products in African countries [12].

Foodborne infection diseases, including campylobacteriosis, affect 10% of the world’s population and cause approximately 33 million deaths each year [13]. *Campylobacter* is a Gram-negative, thermotolerant, and zoonotic bacterial genus that commonly exhibits antibiotic resistance, and it is the leading cause of human gastroenteritis worldwide [14,15]. It is estimated that approximately 20 species have been described within the genus *Campylobacter*. Among these, *Campylobacter jejuni* (*C. jejuni*) and *Campylobacter coli* (*C. coli*) are responsible for the majority of human infections [16]. It is estimated that 80–90% of cases of campylobacteriosis are attributable to *C. jejuni*; meanwhile, 10–20% are caused by *C. coli* [17]. *C. jejuni* has been found in healthy intestines of cattle without clinical symptoms, and it is able to survive under stressful conditions by forming biofilms on surfaces such as milking equipment [18]. *C. coli* has an infectious epidemiology similar to that of *C. jejuni*, with symptoms like diarrhea, abdominal pain, fever, and nausea, among other complications [16]. *C. coli* has become the predominant cause of foodborne illness in developing countries. This is due to its ability to exchange genetic material with other *Campylobacter* species, thereby enhancing its adaptability and pathogenic potential [16,19].

The identification of *Campylobacter* spp. from food samples has traditionally relied on culture methods, including enrichment media, isolation on selective agar, and biochemical identification [20]. There are standardized protocols, such as those set out in ISO 10272-1:2017 and ISO 10272-2:2017, providing internationally accepted guidelines for the detection, enumeration, and confirmation of *Campylobacter* species in food [21,22]. However, these conventional techniques have important limitations, such as the need for long incubation times, the possibility of false negative results due to the viable but non-culturable status of the bacteria, and identification errors caused by microbiological similarities between *Campylobacter* spp., along with other pathogens such as *Arcobacter* spp. or *Helicobacter* spp., which can grow on selective media [23]. In contrast, molecular methods, such as PCR and qPCR, have emerged as superior alternatives due to their higher sensitivity, specificity, and reduced analysis times [24].

The aim of this study is to isolate and molecularly identify *Campylobacter* spp., with focus on *C. jejuni* and *C. coli*, in raw milk samples from Pichincha and Manabí in Ecuador. It is particularly important due to the association between foodborne diseases and raw milk consumption; it is therefore crucial to understand the presence of *Campylobacter* in this product for the purpose of protecting public health. The integration of microbiological, molecular, and statistical analyses enables a more thorough understanding of the risks linked to *Campylobacter* transmission via raw milk.

## 2. Materials and Methods

### 2.1. Sample Collection

Raw milk samples were collected from cattle farm cooling tanks and from popular markets in the country where milk was sold directly to consumers between 5 September 2022, to 16 July 2023, in collaboration with the veterinary faculty of Universidad Central del Ecuador (UCE). The samples were stored in sterile containers and kept at a temperature of 4 °C until they were transferred to the research laboratories of the Universidad de Las Americas (UDLA) for processing. The procedure was carried out in accordance with the guidelines established in NTE INEN-ISO 707 and international standards ISO 7218:2024 [25,26]. The quantity of samples necessitated was calculated based on an expected prevalence of *Campylobacter* spp. of 50%, a predetermined absolute precision of 5%, and a 95% confidence level. The sample size was determined using the standard formula:

(1) $$n=\frac{Z_{2}\times Pexp\times (1-Pexp)}{d_{2}}$$

where *n* is the required sample size, Pexp is the expected prevalence (0.50), d is the desired absolute precision (0.05), and Z is the statistic for a 95% confidence level [27]. It was determined by means of the formula that the minimum number of raw milk samples required to estimate the prevalence of *Campylobacter* spp. was 384 samples. A total of 633 samples were collected in two provinces of Ecuador: Pichincha (*n* = 322) and Manabí (*n* = 311) from small producers (*n* = 246), medium producers (*n* = 207), and large producers (*n* = 180). The classification of each sample was based on its geographical origin (province), climatic condition (warm and rainy), and the size of the producer (Supplementary Material). The classification of producers was determined in accordance with Ministerial Agreement No. 095, which stipulates that small producers are defined as those with between one and fifty head of cattle, medium producers as those with between fifty and two hundred head of cattle, and large producers as those with more than one hundred head of cattle [28]. The climatic conditions indicated have been compiled in accordance with the National Institute of Meteorology and Hydrology (INAMHI). This classifies the period from June to September as a warm season, characterized by high temperatures (20–30 °C) and little rainfall; in contrast, the period from October to May is classified as a rainy season, characterized by lower temperatures (18–22 °C) and constant rainfall. All procedures were carried out in accordance with the regulations and approval of the Committee for the Care and Use of Laboratory Animal Resources and the Agency for Phytosanitary and Zoosanitary Regulation and Control of Ecuador (AGROCALIDAD), as evidenced by authorization number #INT/DA/019.

### 2.2. Milk Enrichment

Enrichment of the samples was achieved by the addition of Brain Heart Infusion (BHI) (Difco™ by Fisher Scientific, Carlsbad, CA 237500, USA) broth in a 9:1 ratio with raw milk, yielding a final volume of 40 mL. Subsequently, the enriched samples were subjected to incubation at 37 °C with shaking at 150× *g* for 24 h.

DNA extraction of pre-enrichment milk samples was performed by subjecting the samples to a thermal shock, which consisted of incubation at −20 °C for 24 h and subsequent heating to 56 °C. Genomic DNA was extracted using the phenol/chloroform method [29].

Briefly, samples were centrifuged at 6000 rpm for 15 min, and the pellet was resuspended in 200 µL of TE buffer. The suspension was frozen at −80 °C for 10 min and then incubated at 56 °C for 1 min. Subsequently, 600 µL of guanidinium thiocyanate reagent was added, followed by gentle inversion and incubation at room temperature for 5 min. DNA was purified by adding 100 µL of chloroform, mixing by inversion, and centrifuging at 12,000× *g* for 5 min at 4 °C. The aqueous phase was transferred to a new tube containing 500 µL of isopropanol, and the mixture was incubated overnight at −20 °C to precipitate the DNA. The pellet was washed three times with 70% ethanol, air-dried, and finally resuspended in 30 µL of TE buffer, then incubated at 56 °C for 15 min.

### 2.3. Bacteria Isolation and DNA Extraction

Enriched samples were inoculated on *Campylobacter* Agar Base (TM Media by Titan Biotech Ltd., Delhi, CA, India), a selective medium for *Campylobacter* spp. accordance with ISO 10272-1:2017 and ISO 10272-2:2017 standards [21,22]. Following this, the samples were subjected to a 24-h incubation period at 37 °C under microaerophilic conditions. Following this incubation, preliminary identification and isolation of *Campylobacter* spp. was determined by the morphology of the colonies, which may present as a flat, grayish growth with irregular borders, or raised, rounded, mucoid-appearing colonies.

For DNA extraction of isolated bacteria, a selection of these colonies was suspended in 200 µL of TE buffer, homogenized using a vortex, and frozen at −20 °C for a period of 24 h. Subsequently, the samples were exposed to a heat treatment at a temperature of 95 °C within a dry bath (Thermo Fisher Scientific, Carlsbad, CA, USA) with gentle agitation. Following a secondary centrifugation under the same conditions as previously outlined, 100 µL of the transparent upper layer was transferred into 200 µL microtubes [30]. The extracted DNA was maintained at a temperature of −20 °C until further molecular analysis.

### 2.4. Molecular Detection of Campylobacter spp. by qPCR

For *Campylobacter* spp. identification, qPCR reactions were employed, utilizing a 10 µL Master Mix consisting of 2X TaqMan™ Universal Master Mix II (Thermo Fisher Scientific, Carlsbad, CA 4364338, USA), 0.2 µM of each primer (Table 1), 0.1 µM of hydrolysis probe (Table 1), and 1 µL of diluted DNA 1:5. The amplification protocol consisted of an initial UNG activation step at 50 °C for 2 min, followed by an initial denaturation at 95 °C for 5 min. Subsequently, 45 amplification cycles were performed, each comprising denaturation at 95 °C for 15 s, annealing and fluorescence reading at 60 °C for 45 s, and extension at 72 °C for 30 s [31]. Amplifications involved the utilization of the CFX96 Touch Real-Time PCR Detection System thermal cycler (Bio-Rad Laboratories, Inc., Hercules, CA 184-5096, USA). A standard curve was generated using genomic DNA from *Campylobacter jejuni* ATCC 33291 as a positive control, using 10-fold serial dilutions between 10^9^ to 1 copy to genetic material. The detection data were analyzed considering positive samples, those that exhibited amplification curves with cycle threshold (Ct) values below the Ct determined for Limit of Detection. This approach allowed a sensitive and specific detection of *Campylobacter* spp. in enriched raw milk samples and confirmation of isolated bacteria samples.

### 2.5. Molecular Identification of C. coli and C. jejuni Using PCR

A duplex PCR assay for the specific identification of *C. jejuni* and *C. coli* was established using previously published pairs of primers for each species (Table 1). The PCR reaction was performed in a final volume of 10 µL, composed of GoTaq^®^ Green Master Mix 2X (Promega Corporation, Wisconsin, CA M7132, USA). The total concentration of each primer was 3 µM, and 1 µL of diluted DNA (1:5) was utilized. PCR amplification was performed with an initial denaturation at 95 °C for 30 s, followed by 40 cycles of denaturation at 94 °C, annealing at 52 °C, and extension at 72 °C [32]. The amplified products were subsequently separated by agarose gel electrophoresis, and DNA fragments were visualized after staining with SYBR Safe DNA Gel Stain (Thermo Fisher Scientific, Carlsbad, CA S33102, USA) using a UV transilluminator (Bio-Rad Laboratories, Inc., Hercules, CA 1708110EDU, USA). On electrophoresis, the size of each fragment was determined by comparing it to a 100-base pair molecular weight marker (Thermo Fisher Scientific, Carlsbad, CA 15628019, USA).

### 2.6. 16S Sequencing and Bioinformatic Analysis

Five samples were randomly selected from each species, in addition to five samples with no identified species; they were used for *16S rDNA* amplification via primers 27F/1492R and GoTaq Green Master Mix (Promega, Madison, WI, USA) [33]. The PCR program included 40 cycles (95 °C, 55 °C, and 72 °C), followed by agarose gel electrophoresis with SYBR Safe staining (Invitrogen by Thermo Fisher Scientific, Carlsbad, CA, USA). Amplicons were then purified using ExoSAP-IT™ (Applied Biosystems, Santa Clara, CA 95051, USA). Purified amplicons were sequenced bidirectionally using BigDye^®^ Terminator v3.1 (Applied Biosystems, Santa Clara, CA, USA). Amplicons were subjected to capillary electrophoresis on an ABI 3500 Genetic Analyzer (Applied Biosystems, Carlsbad, CA, USA). The obtained electropherograms were aligned against a reference sequence using Geneious software v10.2.3 (https://www.geneious.com) and analyzed through BLAST 2.13.0 for sequence identification. The electropherograms were then compared and aligned with other randomly selected sequences that had been deposited in GenBank using the nucleotide similarity matrix with the assistance of Geneious software version 10.2.3 (https://www.geneious.com) (Biomatters Ltd., Auckland, New Zealand). Phylogenetic analysis was performed in MEGA X with the Neighbor-Joining method, applying the p-distance model and 1000 bootstrap replicates to discriminate *Campylobacter* species.

### 2.7. Statistical Analysis

Data from the molecular assays were analyzed using descriptive statistics stratified by province, climatic conditions, and producer size. The prevalence of *Campylobacter* spp. was calculated by the percentage of positive samples in relation to the total number of raw milk samples analyzed. The prevalence of *C. jejuni* and *C. coli* was expressed as the percentage of positive samples for each species among all *Campylobacter*-positive samples [34]. In both cases, prevalence (%) was determined using the following equation:


(2) $$Prevalence(\%)=\frac{Total\;number\;of\;positive\;samples}{Number\;of\;samples\;analyzed}\times 100$$

Before the statistical analyses were conducted, the normality of the data was evaluated using the Shapiro–Wilk test to ascertain whether the dataset followed a normal distribution. The objective of this assessment was to determine the applicability of multivariate tests. The association between the variables of province, climate, and producer size was then evaluated using the Chi-square test (χ^2^). Statistical significance of results was considered at a *p*-value < 0.05; for significant associations, odds ratios (OR) with 95% confidence intervals were calculated. The agreement between qPCR detection and microbiological isolation was evaluated using Cohen’s Kappa coefficient [35]. All statistical tests were carried out using RStudio software version 4.4.3 (https://www.R-project.org/) (R Core Team, Vienna, Austria).

## 3. Results

### 3.1. Molecular Detection of Campylobacter spp. on Enrichment Milk and Isolated Colonies

The qPCR standard curve exhibited high performance, with an amplification efficiency of 96.6% and R^2^ = 0.991, and a limit of detection (LoD) of 1 copy of DNA (Ct = 39) (Supplementary Material). The detection data were analyzed considering positive samples that exhibited amplification curves with cycle threshold (Ct) values below 39. qPCR analysis of pre-enriched raw milk samples revealed the prevalence of *Campylobacter* spp. in 316 of the 633 samples evaluated, resulting in a prevalence of 49.9% (Table 2). On the other hand, microbiological isolation showed a quite similar prevalence of the pathogen in 291 (92.08%) of these samples (Supplementary Material). Cohen’s Kappa coefficient was 0.92, indicating substantial similarity between the two methodologies and suggesting that molecular detection, although very similar, is more sensitive than microbial isolation. A statistically significant association was identified between the province of origin and the prevalence of *Campylobacter* spp. Samples from Manabí had a higher prevalence, seen in 182 samples (57.6%), compared to those from Pichincha (only 134 samples; 42.4%). Chi-square analysis showed a significant association between the province of origin and the prevalence of *Campylobacter* spp. (*p*-value < 0.001), where Manabí presented a higher occurrence of positive samples compared to Pichincha (Supplementary Material). In contrast, the size of the producer did not have a significant influence on the prevalence of *Campylobacter* spp. in the samples analyzed (*p*-value = 0.7932), evidencing similar frequencies among small (*n* = 119), medium (*n* = 104), and large (*n* = 93) producers.

**Figure 1:**
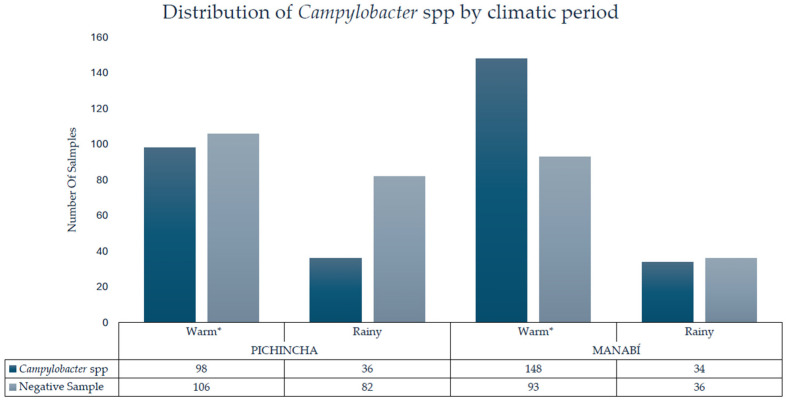
Detection of *Campylobacter* spp. in raw milk by province and climate period; * = statistically significant difference.

The association between the climate season of the samples taken and the detection of *Campylobacter* spp. in the 633 samples of processed raw milk was evaluated. Samples collected during the warm season had a higher percentage of positivity (*n* = 246) compared to those obtained in the rainy season (*n* = 70) (Figure 1). Statistical analysis showed a significant difference between the two periods (*p*-value < 0.001), and the probability of finding *Campylobacter* spp. in raw milk samples was approximately double during the warm season compared to the rainy season (Supplementary Material).

### 3.2. Molecular Identification of C. jejuni and C. coli

In the analysis by province and producer size (Table 3), *C. coli* was the species detected most frequently in all categories, especially in Pichincha, where it reached 50.04% among small producers. In contrast, Manabí had a higher percentage of non-identified *Campylobacter* species. *C. jejuni* was distributed more evenly across provinces and producer sizes, with values ranging from 17.5% to 33.3%. Co-contamination with *C. jejuni* and *C. coli* occurred in all categories, fluctuating between 7.9% and 21.2%. These represent samples that tested positive for both species and are included in their individual counts.

Statistically significant differences were observed between provinces in all categories evaluated (*p*-value < 0.05) (Table 3). Manabí had higher detection rates compared to Pichincha: *C. jejuni* was detected in 13.6% of samples in Manabí, compared to 8.2% in Pichincha; *C. coli* in 30.4% compared to 15. 1%; co-contaminations in 8.6% compared to 4.2%, and unidentified species in 24.5% compared to 17.4%. These findings suggest a higher burden of *Campylobacter* contamination in the production systems of Manabí.

Regarding producer size, a statistically significant difference was detected only for co-contaminations, defined as the simultaneous presence of *C. jejuni* and *C. coli*, (*p* = 0.011). In this instance, samples from small-scale producers were more likely to be contaminated compared to those from larger producers (Supplementary Material). No significant differences were observed for the individual detection of *C. jejuni*, *C. coli*, or non-identified species based on the size of the production unit (*p* > 0.05).

**Figure 2:**
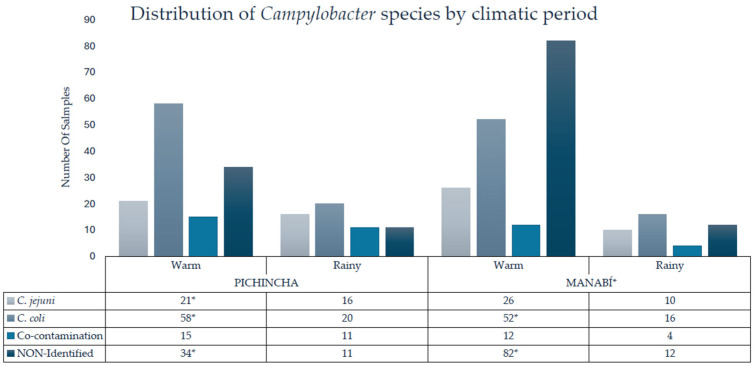
Identification of *C. coli*, *C. jejuni*, co-contamination, and non-identified species in raw milk by province and climate season. Co-contamination was present in samples that tested positive for both species and are included in their individual counts. * = statistically significant difference.

The distribution of *Campylobacter* species showed significant differences between the climate seasons. During warm conditions, *C. jejuni* had a prevalence of 14.9% (47/316), while on rainy seasons it was 8.2% (26/316). *C. coli* was the predominant species in both seasons, with a prevalence of 34.8% (110/316) in warm conditions and 11.4% (36/316) in rainy conditions. Co-contamination reached 8.5% (27/316) during the warm period and 6.6% (21/316) during the rainy period. Non-identified species showed the greatest difference between seasons, with a prevalence of 36.7% (116/316) in warm conditions compared to 7.3% (23/316) in rainy conditions. Statistical analysis using chi-square showed that these variations between periods were significant (*p* = 0.0065), indicating that the climatic period influenced the proportion of *Campylobacter* species detected in raw milk (Figure 2). During warm conditions, most isolates came from Manabí, with *C. coli* being more recurrent in this province (65.1%) than in Pichincha (34.9%); similar with *C. jejuni*, being positive in 59.6% of cases in Manabí. Non-identified strains showed the greatest difference, with 70.6% concentrated in Manabí and only 29.3% in Pichincha, evidencing a higher load and diversity of *Campylobacter* in the coastal region.

### 3.3. 16S Analysis

**Figure 3:**
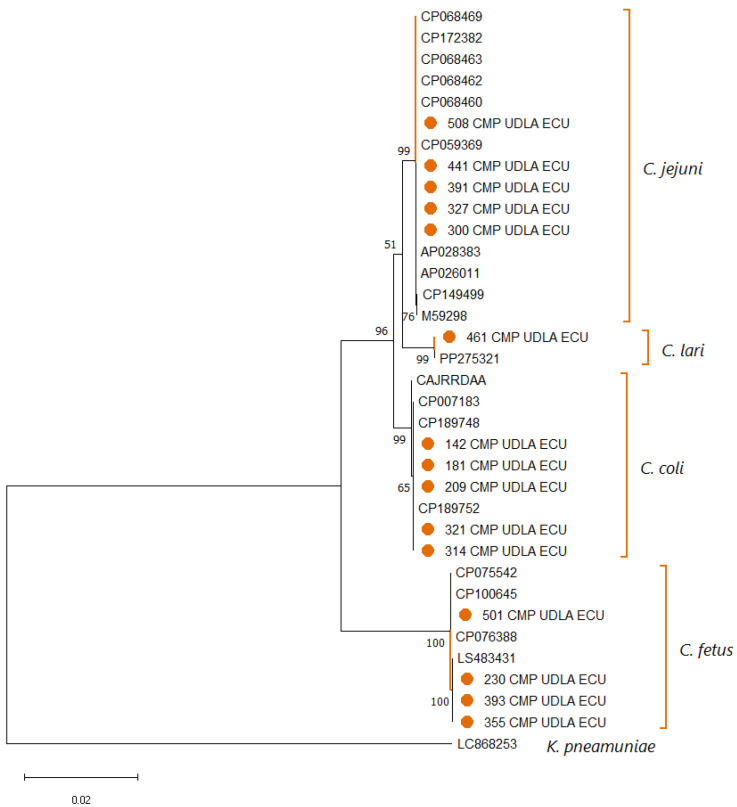
Phylogenetic tree illustrating the relationships among *Campylobacter* spp. sequences obtained in this study and reference sequences retrieved from NCBI, based on partial 16S rDNA gene alignment. Multiple sequence alignment was performed with ClustalX 2.1, and tree construction was carried out using MEGA X. Bootstrap values (1000 replicates) are indicated at branch nodes, and the scale bar denotes the number of substitutions per site. A *Klebsiella pneumoniae* 16S rDNA sequence was included as an outgroup. Sequences generated in this work are highlighted in orange (●).

Analysis of *Campylobacter* 16S sequences showed that for the species *C. coli* and *C. jejuni*, the sequences obtained in this study had NT similarities of 98.5–100% compared to sequences of the same species deposited in GenBank (Supplementary Material). On the other hand, the positive samples for *Campylobacter* spp., not corresponding to those studied in this study, showed a similarity of 100% with sequences of *C. lari* (*n* = 1) and *C. fetus* (*n* = 4) found in GenBank (Supplementary Material). The construction of the phylogenetic tree (Figure 3) resulted in the division of clades for the two species studied (*C. coli* and *C. jejuni*) and the two found in those samples that corresponded to another species of *Campylobacter* (*C. lari* and *C. fetus*).

## 4. Discussion

Raw milk is widely recognized to pose a significant microbiological risk due to its capacity to serve as a vehicle for the transmission of pathogenic microorganisms such as *Campylobacter* spp. Raw milk consumption has frequently been implicated in Campylobacteriosis outbreaks worldwide [36,37]. High incidences of campylobacteriosis have been reported in several countries, including the United States of America, Canada, Mexico, Colombia, and Brazil. In these countries, *C. jejuni* and *C. coli* are frequently ingested by contaminated food [12]. In the present study, a high prevalence of *Campylobacter* spp. was observed in the samples analyzed by qPCR (Table 2), suggesting deficiencies in hygiene and health controls during production [32]. This may indicate a deficiency in regulatory controls due to the possible direct transmission of the pathogen from the animal to the food, given the previously reported correlation between prevalence rates in raw milk and in dairy cow feces [38]. Molecular techniques have established themselves as the most accurate and sensitive methods for detecting foodborne pathogens. DNA detection by qPCR offers up to ten times greater sensitivity than colony-counting methods, such as those established in ISO 7218:2014 [39,40,41]. This methodological advantage is particularly relevant in Ecuador, where limited etiological identification of foodborne infections has led to the non-specific classification of 11,533 cases as “other bacterial infections” between 2015 and 2020 [42]. This need for accurate diagnosis not only prevents the implementation of targeted control measures but can also lead to recurrent outbreaks, ineffective empirical treatment, antimicrobial resistance, and underreporting of emerging zoonotic agents [37].

*Campylobacter* spp. was detected in 49.9% (316/633) of raw milk samples analyzed by qPCR in the provinces of Pichincha and Manabí, Ecuador. This prevalence greatly exceeds that reported in international studies, such as those conducted in Ethiopia, where a 16% prevalence was detected in raw milk, and in Egypt, where a 9.5% prevalence was detected [43]. The differences could be attributed to the greater informality in the Ecuadorian milk production chain, where more than 50% of milk is marketed without adequate regulatory oversight by entities such as the Ecuadorian Agency for Phytosanitary and Zoosanitary Regulation and Control (AGROCALIDAD) [44]. In addition, a local study detected *Campylobacter* spp. in 6.3% of infant diarrhea samples in rural areas of the country, suggesting that transmission could come from a zoonotic source related to food consumption [45,46]. This prevalence is comparable to findings from neighboring countries such as Colombia, where *Campylobacter* spp. was reported in 46.2% of raw milk samples, and is higher than data from southern Peru, where *Campylobacter* accounted for 50.2% of bacterial isolates associated with foodborne diseases in clinical cases between 2006 and 2010 [47,48]. Similarly, studies in Brazil and Chile have demonstrated significant *Campylobacter* contamination in dairy production systems, with reported prevalences of 35.8% and 21.3% in milk from dairy cattle, respectively. These results indicate a persistent regional problem in South America, where deficiencies in hygiene practices, cold chain maintenance, and regulatory oversight contribute to elevated contamination rates [49,50].

Among the species identified, *C. coli* accounted for 46,2% and *C. jejuni* for 23.1% of positive samples. This distribution pattern is the opposite of that observed in studies conducted in India and Portugal, where *C. jejuni* predominated over *C. coli* (58.1% and 30.7%, respectively) [50,51,52,53]. It was similarly observed in other countries where *C. jejuni* predominates; for example, in Brazil, *C. jejuni* accounted for 78.47% of *Campylobacter* isolates from food, while in Colombia, 54.8% were attributed to *C. jejuni and* 9.52% to *C. coli* [47,48]. The higher prevalence in Ecuador could be due to this species’ greater adaptive capacity and resistance, as reflected in its ability to exchange genetic material horizontally and to survive across a range of environmental conditions, including high tropical temperatures and the presence of organic matter [54]. This difference also suggests a distinct ecological profile in the region’s bovine microbiota, possibly influenced by local handling practices [55]. Considering that campylobacteriosis has been the most common gastrointestinal infection in the European Union since 2005, and that Ecuador lacks active epidemiological surveillance for this pathogen, the current findings indicate a latent risk of underdiagnosed outbreaks in the population [56].

A significant association was observed between the province of origin and the detection of *Campylobacter* spp. by qPCR, with Manabí showing the highest prevalence. This result can be explained by the hot, humid climatic conditions of the coastal region, which favor the proliferation of mesophilic bacteria such as *Campylobacter*, and by the lower level of technification of milk production, where small producers still perform manual milking [53,56]. This situation corresponds with reports from the health authority AGROCALIDAD, which show deficiencies in the implementation of Good Livestock Practices in coastal areas [44,57]. Different studies have shown that *Campylobacter* is more common in food from coastal areas, posing a considerable public health risk. In Trinidad, a prevalence of 1.9% was reported, while in Kerala, India, rates of 6.3%, 8.6%, and up to 28.0% were observed in different food products [58,59,60]. These findings demonstrate that *Campylobacter* contamination can reach high levels in warm, humid coastal environments, varying by region and reinforcing the need for active, targeted microbiological surveillance in areas with environmental conditions similar to those of the Ecuadorian coast.

Analysis by producer size showed that small producers had a higher frequency of *Campylobacter* spp. positivity, especially in cases of co-contamination with *C. jejuni* and *C. coli*. This finding is consistent with previous studies indicating that manual milking practices, common in small-scale systems, increase the likelihood of fecal or other organic waste cross-contamination [18]. In contrast, studies in tech systems in Europe report lower contamination rates thanks to the use of automatic milking machines and tank temperature controls [51]. Another study on the detection of zoonotic enteropathogens found co-circulation of *C. jejuni* among domestic animals and children in rural areas of Ecuador. This suggests that zoonotic transmission is facilitated by rural environments without adequate sanitation infrastructure and practices [37,61].

The identification of *Campylobacter jejuni* and *Campylobacter coli* in the analyzed samples is supported by the concordance between results from specific PCR and phylogenetic analysis based on 16S sequences (Figure 3). This double validation reinforces the well-documented role of both species as relevant zoonotic agents, especially in the context of raw milk as a vehicle of transmission [62]. In contrast, the occasional detection of other species of the genus, such as *C. fetus* and *C. lari*, suggests greater microbial diversity than expected, although their direct involvement in human diseases transmitted by milk is not clearly established [63]. These species, although present in certain ecological conditions, are not usually associated with foodborne outbreaks and do not show significant prevalence in surveillance studies [64]. In the future, technologies such as whole-genome sequencing and metagenomic approaches would facilitate the dissemination of knowledge about the genetic characteristics of isolates, including antimicrobial resistance profiles, virulence genes, and other determinants relevant to public health [18]. This type of analysis would provide a comprehensive view of the zoonotic potential and intraspecific diversity of the isolates, while also facilitating the detection of emerging or atypical strains that may escape conventional diagnostic methods; thus supporting a more proactive, prevention-oriented surveillance approach [41].

## 5. Conclusions

This study revealed a high prevalence of the *Campylobacter* genus in raw milk samples from the provinces of Pichincha and Manabí, including the specific detection of *C. jejuni*, *C. coli*, co-contaminations, and unidentified strains. The use of molecular techniques enabled faster, more sensitive pathogen identification than conventional methods. Variations in contamination levels between provinces and farm production size suggest an influence of environmental factors and hygienic practices. The detection of strains not typified by conventional methods underscores the need for more comprehensive diagnostic approaches, such as whole-genome sequencing. Strengthening biosecurity measures at the farm level, combined with continuous monitoring and advanced molecular surveillance, is essential to mitigate the risk of foodborne diseases associated with raw milk consumption.

## Figures and Tables

**Table 1:** Primers and hydrolysis probe used for molecular detection of *Campylobacter* spp. and identification of *C. coli* and *C. jejuni*.

Species	Name	Target	Sequence	Amplicon Length (bp)	Reference
*Campylobacter* spp.	Camp 16S Fwd	*16S rRNA* gene	5′-ACG TGC TAC AAT GGC ATA TAC A-3′	92	[31] Modified
	Camp 16S Rev		5′-CGG CTT CAT GCT CTC GAG T-3′		
	Camp 16S Probe		Cy5.5/5′-TGT CCC AGT TCG GAT TGT TCT CTG C-3′/BHQ-3		
*C. coli*	hipO-F	*hippuricase* gene	5′-GAA GAG GGT TTG GGT GGT G-3′	735	[12]
	hipO-R		5′-AGC TAG CTT CGC ATA ATA ACT TG-3′		
*C. jejuni*	asp-F	*aspartokinase* gene	5′-GGT ATG ATT TCT ACA AAG CGA G-3′	500	[12]
	asp-R		5′-ATA AAA GAC TAT CGT CGC GTG-3′		

**Table 2:** Analysis of *Campylobacter* prevalence by qPCR assay in enriched raw milk samples. Results of positive samples for *Campylobacter* spp. are presented according to province and producer size.

Province	Producer Size	*Campylobacter* spp.	Negative Sample	Total Sample
**PICHINCHA**	**Small**	56 (43.4%)	73	129
	**Medium**	45 (41.7%)	63	108
	**Large**	33 (38.8%)	52	85
MANABÍ *	**Small**	63 (53.8%)	54	117
	**Medium**	59 (59.6%)	40	99
	**Large**	60 (63.2%)	35	95
**Total Positive**		316 (49.9%)	317	633

* = statistically significant difference.

**Table 3:** Distribution of *Campylobacter* species detected by PCR in positive raw milk samples by province and producer size. Co-contamination represents samples that tested positive for both species and are included in their individual counts.

Province	Producer Size	*C. jejuni*	*C. coli*	Co-Contamination	NON-Identified	Total Sample
**PICHINCHA**	**Small**	13 (23.2%)	33 (58.9%) *	10 (17.9%) *	20 (35.7%)	56
	**Medium**	15 (33.3%)	24 (53.3%)	9 (20.0%)	15 (33.3%)	45
	**Large**	9 (27.3%)	21 (63.6%)	7 (21.2%)	10 (30.3%)	33
MANABÍ *	**Small**	11 (17.5%)	27 (42.9%)	5 (7.9%)	30 (47.6%)	63
	**Medium**	11 (18.6%)	24 (40.7%)	5 (8.5%)	29 (49.2%)	59
	**Large**	14 (23.3%)	17 (28.3%)	6 (10.0%)	35 (58.3%)	60
**Total Positive**		73	146	42	139	316

* = statistically significant difference.

## Data Availability

The original contributions of this study are included in the article/Supplementary Materials; further inquiries can be directed to the corresponding author.

## Supplementary Materials

The following supporting information can be downloaded at: https://www.mdpi.com/article/10.3390/pathogens14111155/s1, Figure S1: Standard curve; Table S1: Nucleotide Matrix; Table S2: Complete Dataset.

## Abbreviations

The following abbreviations are used in this manuscript:

AGROCALIDAD	Agency for Phytosanitary and Zoosanitary Regulation and Control of Ecuador
ISO	International Organization for Standardization
INEN	Ecuadorian Standardization Service
INAMHI	National Institute of Meteorology and Hydrology
UCE	Universidad Central del Ecuador
UDLA	Universidad de las Americas
*C. coli*	*Campylobacter coli*
*C. jejuni*	*Campylobacter jejuni*
*C. fetus*	*Campylobacter fetus*
*C. lari*	*Campylobacter lari*
LoD	Limit of Detection
Ct	Cycle Threshold
