# Supplementary material for: Raw Milk as a Source of Campylobacter Infection: Isolation and Molecular Identification of Campylobacter coli and Campylobacter jejuni in Ecuador
Source: Pathogens. 2025 Nov 13;14(11):1155. doi: 10.3390/pathogens14111155 (PMC12655607; doi:10.3390/pathogens14111155)
Supplement: Supplementary file 1 [file pathogens-14-01155-s001.zip › pathogens-3942722-supplementary.pdf]

Supplementary Material

Figure S1 - Standard Curve

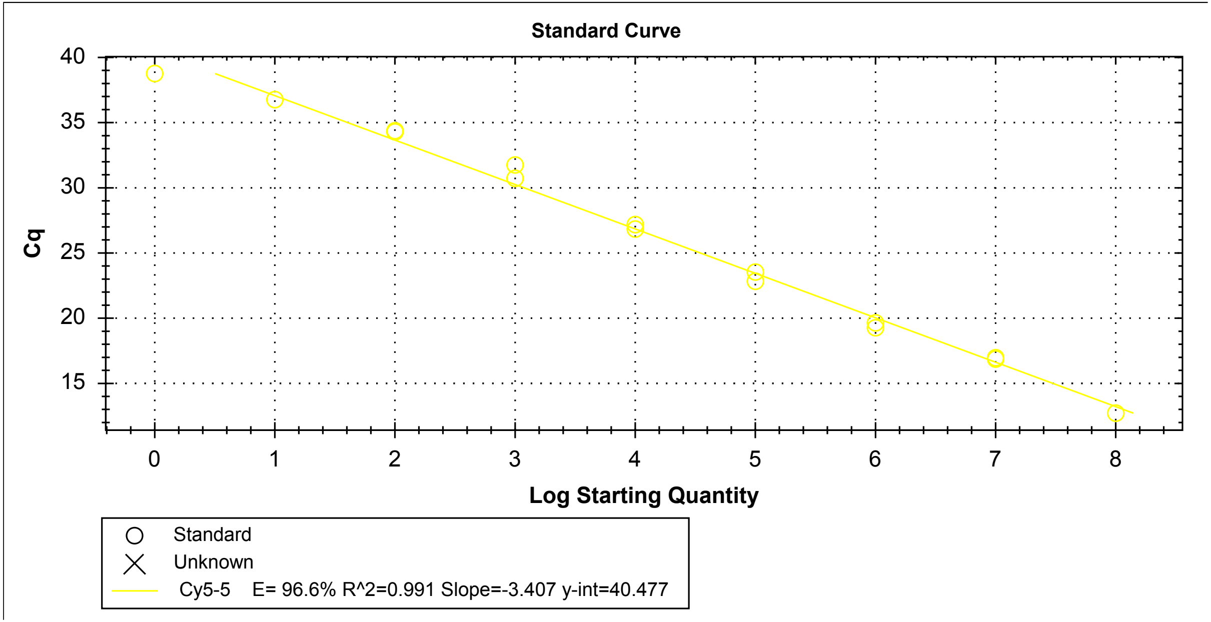

Table S1 - Nucleotide Matrix

SAMPLE ID	Percentage of nucleotide identity between the *Campylobacter* sequences of Ecuador and sequences from other countries																																		
	LC868253	CP075542	CP100645	501 CMP UDLA ECU	CP076388	LS483431	230 CMP UDLA ECU	393 CMP UDLA ECU	355 CMP UDLA ECU	CAJRRDAA	CP007183	CP189748	142 CMP UDLA ECU	181 CMP UDLA ECU	209 CMP UDLA ECU	CP189752	321 CMP UDLA ECU	314 CMP UDLA ECU	461 CMP UDLA ECU	PP275321	AP026011	AP028383	300 CMP UDLA ECU	327 CMP UDLA ECU	391 CMP UDLA ECU	441 CMP UDLA ECU	CP059369	508 CMP UDLA ECU	CP068460	CP068462	CP068463	CP068469	CP172382	CP149499	M59298
LC868253		72,809%	72,809%	72,809%	72,809%	72,879%	72,879%	72,879%	72,879%	72,946%	73,538%	73,538%	73,538%	73,538%	73,538%	73,538%	73,538%	73,538%	73,886%	73,886%	73,955%	73,955%	73,955%	73,955%	73,955%	73,955%	73,955%	73,955%	73,955%	73,955%	73,955%	73,955%	73,955%	73,955%	73,433%
CP075542	72,809%		100%	100%	100%	99,932%	99,932%	99,932%	99,932%	93,938%	94,384%	94,384%	94,384%	94,384%	94,384%	94,384%	94,384%	94,384%	93,288%	93,288%	93,375%	93,375%	93,375%	93,375%	93,375%	93,375%	93,375%	93,375%	93,375%	93,375%	93,375%	93,375%	93,375%	93,306%	92,185%
CP100645	72,809%	100%		100%	100%	99,932%	99,932%	99,932%	99,932%	93,938%	94,384%	94,384%	94,384%	94,384%	94,384%	94,384%	94,384%	94,384%	93,288%	93,288%	93,375%	93,375%	93,375%	93,375%	93,375%	93,375%	93,375%	93,375%	93,375%	93,375%	93,375%	93,375%	93,375%	93,306%	92,185%
501 CMP UDLA ECU	72,809%	100%	100%		100%	99,932%	99,932%	99,932%	99,932%	93,938%	94,384%	94,384%	94,384%	94,384%	94,384%	94,384%	94,384%	94,384%	93,288%	93,288%	93,375%	93,375%	93,375%	93,375%	93,375%	93,375%	93,375%	93,375%	93,375%	93,375%	93,375%	93,375%	93,375%	93,306%	92,185%
CP076388	72,809%	100%	100%	100%		99,932%	99,932%	99,932%	99,932%	93,938%	94,384%	94,384%	94,384%	94,384%	94,384%	94,384%	94,384%	94,384%	93,288%	93,288%	93,375%	93,375%	93,375%	93,375%	93,375%	93,375%	93,375%	93,375%	93,375%	93,375%	93,375%	93,375%	93,375%	93,306%	92,185%
LS483431	72,879%	99,932%	99,932%	99,932%	99,932%		100%	100%	100%	93,870%	94,315%	94,315%	94,315%	94,315%	94,315%	94,315%	94,315%	94,315%	93,219%	93,219%	93,306%	93,306%	93,306%	93,306%	93,306%	93,306%	93,306%	93,306%	93,306%	93,306%	93,306%	93,306%	93,306%	93,237%	92,117%
230 CMP UDLA ECU	72,879%	99,932%	99,932%	99,932%	99,932%	100%		100%	100%	93,870%	94,315%	94,315%	94,315%	94,315%	94,315%	94,315%	94,315%	94,315%	93,219%	93,219%	93,306%	93,306%	93,306%	93,306%	93,306%	93,306%	93,306%	93,306%	93,306%	93,306%	93,306%	93,306%	93,306%	93,237%	92,117%
393 CMP UDLA ECU	72,879%	99,932%	99,932%	99,932%	99,932%	100%	100%		100%	93,870%	94,315%	94,315%	94,315%	94,315%	94,315%	94,315%	94,315%	94,315%	93,219%	93,219%	93,306%	93,306%	93,306%	93,306%	93,306%	93,306%	93,306%	93,306%	93,306%	93,306%	93,306%	93,306%	93,306%	93,237%	92,117%
355 CMP UDLA ECU	72,879%	99,932%	99,932%	99,932%	99,932%	100%	100%	100%		93,870%	94,315%	94,315%	94,315%	94,315%	94,315%	94,315%	94,315%	94,315%	93,219%	93,219%	93,306%	93,306%	93,306%	93,306%	93,306%	93,306%	93,306%	93,306%	93,306%	93,306%	93,306%	93,306%	93,306%	93,237%	92,117%
CAJRRDAA	72,946%	93,938%	93,938%	93,938%	93,938%	93,870%	93,870%	93,870%	93,870%		99,417%	99,417%	99,417%	99,417%	99,417%	99,417%	99,417%	99,417%	96,948%	96,948%	98,098%	98,098%	98,098%	98,098%	98,098%	98,098%	98,098%	98,098%	98,098%	98,098%	98,098%	98,098%	98,098%	98,029%	96,967%
CP007183	73,538%	94,384%	94,384%	94,384%	94,384%	94,315%	94,315%	94,315%	94,315%	99,417%		100%	100%	100%	100%	100%	100%	100%	97,394%	97,394%	98,548%	98,548%	98,548%	98,548%	98,548%	98,548%	98,548%	98,548%	98,548%	98,548%	98,548%	98,548%	98,548%	98,548%	97,378%
CP189748	73,538%	94,384%	94,384%	94,384%	94,384%	94,315%	94,315%	94,315%	94,315%	99,417%	100%		100%	100%	100%	100%	100%	100%	97,394%	97,394%	98,548%	98,548%	98,548%	98,548%	98,548%	98,548%	98,548%	98,548%	98,548%	98,548%	98,548%	98,548%	98,548%	98,548%	97,378%
142 CMP UDLA ECU	73,538%	94,384%	94,384%	94,384%	94,384%	94,315%	94,315%	94,315%	94,315%	99,417%	100%	100%		100%	100%	100%	100%	100%	97,394%	97,394%	98,548%	98,548%	98,548%	98,548%	98,548%	98,548%	98,548%	98,548%	98,548%	98,548%	98,548%	98,548%	98,548%	98,548%	97,378%
181 CMP UDLA ECU	73,538%	94,384%	94,384%	94,384%	94,384%	94,315%	94,315%	94,315%	94,315%	99,417%	100%	100%	100%		100%	100%	100%	100%	97,394%	97,394%	98,548%	98,548%	98,548%	98,548%	98,548%	98,548%	98,548%	98,548%	98,548%	98,548%	98,548%	98,548%	98,548%	98,548%	97,378%
209 CMP UDLA ECU	73,538%	94,384%	94,384%	94,384%	94,384%	94,315%	94,315%	94,315%	94,315%	99,417%	100%	100%	100%	100%		100%	100%	100%	97,394%	97,394%	98,548%	98,548%	98,548%	98,548%	98,548%	98,548%	98,548%	98,548%	98,548%	98,548%	98,548%	98,548%	98,548%	98,548%	97,378%
CP189752	73,538%	94,384%	94,384%	94,384%	94,384%	94,315%	94,315%	94,315%	94,315%	99,417%	100%	100%	100%	100%	100%		100%	100%	97,394%	97,394%	98,548%	98,548%	98,548%	98,548%	98,548%	98,548%	98,548%	98,548%	98,548%	98,548%	98,548%	98,548%	98,548%	98,548%	97,378%
321 CMP UDLA ECU	73,538%	94,384%	94,384%	94,384%	94,384%	94,315%	94,315%	94,315%	94,315%	99,417%	100%	100%	100%	100%	100%	100%		100%	97,394%	97,394%	98,548%	98,548%	98,548%	98,548%	98,548%	98,548%	98,548%	98,548%	98,548%	98,548%	98,548%	98,548%	98,548%	98,548%	97,378%
314 CMP UDLA ECU	73,538%	94,384%	94,384%	94,384%	94,384%	94,315%	94,315%	94,315%	94,315%	99,417%	100%	100%	100%	100%	100%	100%	100%		97,394%	97,394%	98,548%	98,548%	98,548%	98,548%	98,548%	98,548%	98,548%	98,548%	98,548%	98,548%	98,548%	98,548%	98,548%	98,548%	97,378%
461 CMP UDLA ECU	73,886%	93,288%	93,288%	93,288%	93,288%	93,219%	93,219%	93,219%	93,219%	96,948%	97,394%	97,394%	97,394%	97,394%	97,394%	97,394%	97,394%	97,394%		100%	98,409%	98,409%	98,409%	98,409%	98,409%	98,409%	98,409%	98,409%	98,409%	98,409%	98,409%	98,409%	98,409%	98,340%	97,173%
PP275321	73,886%	93,288%	93,288%	93,288%	93,288%	93,219%	93,219%	93,219%	93,219%	96,948%	97,394%	97,394%	97,394%	97,394%	97,394%	97,394%	97,394%	97,394%	100%		98,409%	98,409%	98,409%	98,409%	98,409%	98,409%	98,409%	98,409%	98,409%	98,409%	98,409%	98,409%	98,409%	98,340%	97,173%
AP026011	73,955%	93,375%	93,375%	93,375%	93,375%	93,306%	93,306%	93,306%	93,306%	98,098%	98,548%	98,548%	98,548%	98,548%	98,548%	98,548%	98,548%	98,548%	98,409%	98,409%		100%	100%	100%	100%	100%	100%	100%	100%	100%	100%	100%	100%	99,931%	99,051%
AP028383	73,955%	93,375%	93,375%	93,375%	93,375%	93,306%	93,306%	93,306%	93,306%	98,098%	98,548%	98,548%	98,548%	98,548%	98,548%	98,548%	98,548%	98,548%	98,409%	98,409%	100%		100%	100%	100%	100%	100%	100%	100%	100%	100%	100%	100%	99,931%	99,051%
300 CMP UDLA ECU	73,955%	93,375%	93,375%	93,375%	93,375%	93,306%	93,306%	93,306%	93,306%	98,098%	98,548%	98,548%	98,548%	98,548%	98,548%	98,548%	98,548%	98,548%	98,409%	98,409%	100%	100%		100%	100%	100%	100%	100%	100%	100%	100%	100%	100%	99,931%	99,051%
327 CMP UDLA ECU	73,955%	93,375%	93,375%	93,375%	93,375%	93,306%	93,306%	93,306%	93,306%	98,098%	98,548%	98,548%	98,548%	98,548%	98,548%	98,548%	98,548%	98,548%	98,409%	98,409%	100%	100%	100%		100%	100%	100%	100%	100%	100%	100%	100%	100%	99,931%	99,051%
391 CMP UDLA ECU	73,955%	93,375%	93,375%	93,375%	93,375%	93,306%	93,306%	93,306%	93,306%	98,098%	98,548%	98,548%	98,548%	98,548%	98,548%	98,548%	98,548%	98,548%	98,409%	98,409%	100%	100%	100%	100%		100%	100%	100%	100%	100%	100%	100%	100%	99,931%	99,051%
441 CMP UDLA ECU	73,955%	93,375%	93,375%	93,375%	93,375%	93,306%	93,306%	93,306%	93,306%	98,098%	98,548%	98,548%	98,548%	98,548%	98,548%	98,548%	98,548%	98,548%	98,409%	98,409%	100%	100%	100%	100%	100%		100%	100%	100%	100%	100%	100%	100%	99,931%	99,051%
CP059369	73,955%	93,375%	93,375%	93,375%	93,375%	93,306%	93,306%	93,306%	93,306%	98,098%	98,548%	98,548%	98,548%	98,548%	98,548%	98,548%	98,548%	98,548%	98,409%	98,409%	100%	100%	100%	100%	100%	100%		100%	100%	100%	100%	100%	100%	99,931%	99,051%
508 CMP UDLA ECU	73,955%	93,375%	93,375%	93,375%	93,375%	93,306%	93,306%	93,306%	93,306%	98,098%	98,548%	98,548%	98,548%	98,548%	98,548%	98,548%	98,548%	98,548%	98,409%	98,409%	100%	100%	100%	100%	100%	100%	100%		100%	100%	100%	100%	100%	99,931%	99,051%
CP068460	73,955%	93,375%	93,375%	93,375%	93,375%	93,306%	93,306%	93,306%	93,306%	98,098%	98,548%	98,548%	98,548%	98,548%	98,548%	98,548%	98,548%	98,548%	98,409%	98,409%	100%	100%	100%	100%	100%	100%	100%	100%		100%	100%	100%	100%	99,931%	99,051%
CP068462	73,955%	93,375%	93,375%	93,375%	93,375%	93,306%	93,306%	93,306%	93,306%	98,098%	98,548%	98,548%	98,548%	98,548%	98,548%	98,548%	98,548%	98,548%	98,409%	98,409%	100%	100%	100%	100%	100%	100%	100%	100%	100%		100%	100%	100%	99,931%	99,051%
CP068463	73,955%	93,375%	93,375%	93,375%	93,375%	93,306%	93,306%	93,306%	93,306%	98,098%	98,548%	98,548%	98,548%	98,548%	98,548%	98,548%	98,548%	98,548%	98,409%	98,409%	100%	100%	100%	100%	100%	100%	100%	100%	100%	100%		100%	100%	99,931%	99,051%
CP068469	73,955%	93,375%	93,375%	93,375%	93,375%	93,306%	93,306%	93,306%	93,306%	98,098%	98,548%	98,548%	98,548%	98,548%	98,548%	98,548%	98,548%	98,548%	98,409%	98,409%	100%	100%	100%	100%	100%	100%	100%	100%	100%	100%	100%		100%	99,931%	99,051%
CP172382	73,955%	93,375%	93,375%	93,375%	93,375%	93,306%	93,306%	93,306%	93,306%	98,098%	98,548%	98,548%	98,548%	98,548%	98,548%	98,548%	98,548%	98,548%	98,409%	98,409%	100%	100%	100%	100%	100%	100%	100%	100%	100%	100%	100%	100%		99,931%	99,051%
CP149499	73,955%	93,306%	93,306%	93,306%	93,306%	93,237%	93,237%	93,237%	93,237%	98,029%	98,548%	98,548%	98,548%	98,548%	98,548%	98,548%	98,548%	98,548%	98,340%	98,340%	99,931%	99,931%	99,931%	99,931%	99,931%	99,931%	99,931%	99,931%	99,931%	99,931%	99,931%	99,931%	99,931%		99,051%
M59298	73,433%	92,185%	92,185%	92,185%	92,185%	92,117%	92,117%	92,117%	92,117%	96,967%	97,378%	97,378%	97,378%	97,378%	97,378%	97,378%	97,378%	97,378%	97,173%	97,173%	99,051%	99,051%	99,051%	99,051%	99,051%	99,051%	99,051%	99,051%	99,051%	99,051%	99,051%	99,051%	99,051%	99,051%	

Percentage of nucleotide identity between the Campylobacter sequences of Ecuador and sequences from other countries

Table S2 - Complete Dataset

SAMPLE	PROVINCE	PRODUCER SIZE	CLIMATIC PERIOD	Molecular Detection			Co-contamination (Samples that tested positive for both species and are included in their individual counts)	NON-Identified (Another species of *Campylobacter*)	Isolation
				qPCR	PCR				
				Campylobacter spp	*C. jejuni*	*C. coli*			*Campylobacter* spp
1	PICHINCHA	SMALL	WARM	NEGATIVE	NEGATIVE	NEGATIVE	NEGATIVE	NEGATIVE	NEGATIVE
2	PICHINCHA	SMALL	WARM	NEGATIVE	NEGATIVE	NEGATIVE	NEGATIVE	NEGATIVE	NEGATIVE
3	PICHINCHA	MEDIUM	WARM	NEGATIVE	NEGATIVE	NEGATIVE	NEGATIVE	NEGATIVE	NEGATIVE
4	PICHINCHA	SMALL	WARM	NEGATIVE	NEGATIVE	NEGATIVE	NEGATIVE	NEGATIVE	NEGATIVE
5	PICHINCHA	SMALL	WARM	POSITIVE	NEGATIVE	POSITIVE	NEGATIVE	NEGATIVE	POSITIVE
6	PICHINCHA	SMALL	WARM	NEGATIVE	NEGATIVE	NEGATIVE	NEGATIVE	NEGATIVE	NEGATIVE
7	PICHINCHA	SMALL	WARM	NEGATIVE	NEGATIVE	NEGATIVE	NEGATIVE	NEGATIVE	NEGATIVE
8	PICHINCHA	SMALL	WARM	POSITIVE	NEGATIVE	NEGATIVE	NEGATIVE	POSITIVE	POSITIVE
9	PICHINCHA	MEDIUM	WARM	NEGATIVE	NEGATIVE	NEGATIVE	NEGATIVE	NEGATIVE	NEGATIVE
10	PICHINCHA	SMALL	WARM	POSITIVE	NEGATIVE	POSITIVE	NEGATIVE	NEGATIVE	POSITIVE
11	PICHINCHA	SMALL	WARM	NEGATIVE	NEGATIVE	NEGATIVE	NEGATIVE	NEGATIVE	NEGATIVE
12	PICHINCHA	SMALL	WARM	POSITIVE	POSITIVE	POSITIVE	POSITIVE	NEGATIVE	POSITIVE
13	PICHINCHA	MEDIUM	WARM	NEGATIVE	NEGATIVE	NEGATIVE	NEGATIVE	NEGATIVE	NEGATIVE
14	PICHINCHA	SMALL	WARM	POSITIVE	NEGATIVE	POSITIVE	NEGATIVE	NEGATIVE	POSITIVE
15	PICHINCHA	SMALL	WARM	NEGATIVE	NEGATIVE	NEGATIVE	NEGATIVE	NEGATIVE	NEGATIVE
16	PICHINCHA	MEDIUM	WARM	NEGATIVE	NEGATIVE	NEGATIVE	NEGATIVE	NEGATIVE	NEGATIVE
17	PICHINCHA	MEDIUM	WARM	NEGATIVE	NEGATIVE	NEGATIVE	POSITIVE	NEGATIVE	NEGATIVE
18	PICHINCHA	SMALL	WARM	POSITIVE	NEGATIVE	POSITIVE	NEGATIVE	NEGATIVE	POSITIVE
19	PICHINCHA	MEDIUM	WARM	NEGATIVE	NEGATIVE	NEGATIVE	NEGATIVE	NEGATIVE	NEGATIVE
20	PICHINCHA	MEDIUM	WARM	NEGATIVE	NEGATIVE	NEGATIVE	NEGATIVE	NEGATIVE	NEGATIVE
21	PICHINCHA	SMALL	WARM	NEGATIVE	NEGATIVE	NEGATIVE	NEGATIVE	NEGATIVE	NEGATIVE
22	PICHINCHA	SMALL	WARM	NEGATIVE	NEGATIVE	NEGATIVE	NEGATIVE	NEGATIVE	NEGATIVE
23	PICHINCHA	SMALL	WARM	NEGATIVE	NEGATIVE	NEGATIVE	NEGATIVE	NEGATIVE	NEGATIVE
24	PICHINCHA	MEDIUM	WARM	POSITIVE	NEGATIVE	POSITIVE	NEGATIVE	NEGATIVE	POSITIVE

25	PICHINCHA	SMALL	WARM	NEGATIVE	NEGATIVE	NEGATIVE	NEGATIVE	NEGATIVE	NEGATIVE
26	PICHINCHA	LARGE	WARM	NEGATIVE	NEGATIVE	NEGATIVE	NEGATIVE	NEGATIVE	NEGATIVE
27	PICHINCHA	LARGE	WARM	POSITIVE	NEGATIVE	POSITIVE	NEGATIVE	NEGATIVE	POSITIVE
28	PICHINCHA	SMALL	WARM	NEGATIVE	NEGATIVE	NEGATIVE	NEGATIVE	NEGATIVE	NEGATIVE
29	PICHINCHA	MEDIUM	WARM	NEGATIVE	NEGATIVE	NEGATIVE	NEGATIVE	NEGATIVE	NEGATIVE
30	PICHINCHA	LARGE	WARM	NEGATIVE	NEGATIVE	NEGATIVE	NEGATIVE	NEGATIVE	NEGATIVE
31	PICHINCHA	MEDIUM	WARM	NEGATIVE	NEGATIVE	NEGATIVE	NEGATIVE	NEGATIVE	NEGATIVE
32	PICHINCHA	LARGE	WARM	NEGATIVE	NEGATIVE	NEGATIVE	NEGATIVE	NEGATIVE	NEGATIVE
33	PICHINCHA	SMALL	WARM	NEGATIVE	NEGATIVE	NEGATIVE	NEGATIVE	NEGATIVE	NEGATIVE
34	PICHINCHA	MEDIUM	WARM	NEGATIVE	NEGATIVE	NEGATIVE	NEGATIVE	NEGATIVE	NEGATIVE
35	PICHINCHA	SMALL	WARM	NEGATIVE	NEGATIVE	NEGATIVE	NEGATIVE	NEGATIVE	NEGATIVE
36	PICHINCHA	LARGE	WARM	NEGATIVE	NEGATIVE	NEGATIVE	NEGATIVE	NEGATIVE	NEGATIVE
37	PICHINCHA	MEDIUM	WARM	NEGATIVE	NEGATIVE	NEGATIVE	NEGATIVE	NEGATIVE	NEGATIVE
38	PICHINCHA	LARGE	WARM	NEGATIVE	NEGATIVE	NEGATIVE	NEGATIVE	NEGATIVE	NEGATIVE
39	PICHINCHA	SMALL	WARM	NEGATIVE	NEGATIVE	NEGATIVE	NEGATIVE	NEGATIVE	NEGATIVE
40	PICHINCHA	SMALL	WARM	POSITIVE	NEGATIVE	POSITIVE	NEGATIVE	NEGATIVE	POSITIVE
41	PICHINCHA	MEDIUM	WARM	NEGATIVE	NEGATIVE	NEGATIVE	NEGATIVE	NEGATIVE	NEGATIVE
42	PICHINCHA	SMALL	WARM	NEGATIVE	NEGATIVE	NEGATIVE	NEGATIVE	NEGATIVE	NEGATIVE
43	PICHINCHA	SMALL	WARM	NEGATIVE	NEGATIVE	NEGATIVE	NEGATIVE	NEGATIVE	NEGATIVE
44	PICHINCHA	SMALL	WARM	NEGATIVE	NEGATIVE	NEGATIVE	NEGATIVE	NEGATIVE	NEGATIVE
45	PICHINCHA	MEDIUM	WARM	NEGATIVE	NEGATIVE	NEGATIVE	NEGATIVE	NEGATIVE	NEGATIVE
46	PICHINCHA	MEDIUM	RAINY	NEGATIVE	NEGATIVE	NEGATIVE	NEGATIVE	NEGATIVE	NEGATIVE
47	PICHINCHA	MEDIUM	RAINY	NEGATIVE	NEGATIVE	NEGATIVE	NEGATIVE	NEGATIVE	NEGATIVE
48	PICHINCHA	MEDIUM	RAINY	NEGATIVE	NEGATIVE	NEGATIVE	NEGATIVE	NEGATIVE	NEGATIVE
49	PICHINCHA	MEDIUM	RAINY	NEGATIVE	NEGATIVE	NEGATIVE	NEGATIVE	NEGATIVE	NEGATIVE
50	PICHINCHA	MEDIUM	RAINY	NEGATIVE	NEGATIVE	NEGATIVE	NEGATIVE	NEGATIVE	NEGATIVE
51	PICHINCHA	SMALL	RAINY	NEGATIVE	NEGATIVE	NEGATIVE	NEGATIVE	NEGATIVE	NEGATIVE
52	PICHINCHA	SMALL	RAINY	POSITIVE	NEGATIVE	NEGATIVE	NEGATIVE	POSITIVE	POSITIVE
53	PICHINCHA	MEDIUM	RAINY	POSITIVE	NEGATIVE	NEGATIVE	NEGATIVE	POSITIVE	POSITIVE
54	PICHINCHA	LARGE	RAINY	POSITIVE	NEGATIVE	NEGATIVE	NEGATIVE	POSITIVE	POSITIVE
55	PICHINCHA	MEDIUM	RAINY	NEGATIVE	NEGATIVE	NEGATIVE	NEGATIVE	NEGATIVE	NEGATIVE

56	PICHINCHA	LARGE	RAINY	POSITIVE	NEGATIVE	POSITIVE	NEGATIVE	NEGATIVE	POSITIVE
57	PICHINCHA	MEDIUM	RAINY	NEGATIVE	NEGATIVE	NEGATIVE	NEGATIVE	NEGATIVE	NEGATIVE
58	PICHINCHA	MEDIUM	RAINY	POSITIVE	NEGATIVE	NEGATIVE	NEGATIVE	POSITIVE	POSITIVE
59	PICHINCHA	SMALL	RAINY	NEGATIVE	NEGATIVE	NEGATIVE	NEGATIVE	NEGATIVE	NEGATIVE
60	PICHINCHA	MEDIUM	RAINY	POSITIVE	NEGATIVE	NEGATIVE	NEGATIVE	POSITIVE	POSITIVE
61	PICHINCHA	SMALL	RAINY	NEGATIVE	NEGATIVE	NEGATIVE	NEGATIVE	NEGATIVE	NEGATIVE
62	PICHINCHA	LARGE	RAINY	POSITIVE	NEGATIVE	POSITIVE	NEGATIVE	NEGATIVE	POSITIVE
63	PICHINCHA	MEDIUM	RAINY	NEGATIVE	NEGATIVE	NEGATIVE	NEGATIVE	NEGATIVE	NEGATIVE
64	PICHINCHA	SMALL	RAINY	NEGATIVE	NEGATIVE	NEGATIVE	NEGATIVE	NEGATIVE	NEGATIVE
65	PICHINCHA	MEDIUM	RAINY	NEGATIVE	NEGATIVE	NEGATIVE	NEGATIVE	NEGATIVE	NEGATIVE
66	MANABÍ	LARGE	RAINY	POSITIVE	NEGATIVE	NEGATIVE	NEGATIVE	POSITIVE	POSITIVE
67	MANABÍ	MEDIUM	RAINY	NEGATIVE	NEGATIVE	NEGATIVE	NEGATIVE	NEGATIVE	NEGATIVE
68	MANABÍ	MEDIUM	RAINY	POSITIVE	NEGATIVE	POSITIVE	NEGATIVE	NEGATIVE	POSITIVE
69	MANABÍ	LARGE	RAINY	NEGATIVE	NEGATIVE	NEGATIVE	NEGATIVE	NEGATIVE	NEGATIVE
70	MANABÍ	SMALL	RAINY	POSITIVE	NEGATIVE	NEGATIVE	NEGATIVE	POSITIVE	POSITIVE
71	MANABÍ	LARGE	RAINY	NEGATIVE	NEGATIVE	NEGATIVE	NEGATIVE	NEGATIVE	NEGATIVE
72	MANABÍ	LARGE	RAINY	POSITIVE	NEGATIVE	NEGATIVE	NEGATIVE	POSITIVE	POSITIVE
73	MANABÍ	MEDIUM	RAINY	POSITIVE	POSITIVE	POSITIVE	POSITIVE	NEGATIVE	POSITIVE
74	MANABÍ	SMALL	RAINY	POSITIVE	NEGATIVE	NEGATIVE	NEGATIVE	POSITIVE	POSITIVE
75	MANABÍ	LARGE	RAINY	POSITIVE	NEGATIVE	NEGATIVE	NEGATIVE	POSITIVE	POSITIVE
76	PICHINCHA	MEDIUM	RAINY	POSITIVE	POSITIVE	NEGATIVE	NEGATIVE	NEGATIVE	POSITIVE
77	PICHINCHA	MEDIUM	RAINY	POSITIVE	POSITIVE	POSITIVE	POSITIVE	NEGATIVE	POSITIVE
78	PICHINCHA	MEDIUM	RAINY	NEGATIVE	NEGATIVE	NEGATIVE	NEGATIVE	NEGATIVE	NEGATIVE
79	PICHINCHA	LARGE	RAINY	POSITIVE	POSITIVE	POSITIVE	POSITIVE	NEGATIVE	POSITIVE
80	PICHINCHA	LARGE	RAINY	NEGATIVE	NEGATIVE	NEGATIVE	NEGATIVE	NEGATIVE	NEGATIVE
81	PICHINCHA	SMALL	RAINY	NEGATIVE	NEGATIVE	NEGATIVE	NEGATIVE	NEGATIVE	NEGATIVE
82	PICHINCHA	MEDIUM	RAINY	POSITIVE	POSITIVE	POSITIVE	POSITIVE	NEGATIVE	POSITIVE
83	PICHINCHA	MEDIUM	RAINY	NEGATIVE	NEGATIVE	NEGATIVE	NEGATIVE	NEGATIVE	NEGATIVE
84	PICHINCHA	LARGE	RAINY	NEGATIVE	NEGATIVE	NEGATIVE	NEGATIVE	NEGATIVE	NEGATIVE
85	PICHINCHA	MEDIUM	RAINY	NEGATIVE	NEGATIVE	NEGATIVE	NEGATIVE	NEGATIVE	NEGATIVE
86	PICHINCHA	LARGE	RAINY	POSITIVE	NEGATIVE	POSITIVE	NEGATIVE	NEGATIVE	POSITIVE

87	PICHINCHA	SMALL	RAINY	NEGATIVE	NEGATIVE	NEGATIVE	NEGATIVE	NEGATIVE	NEGATIVE
88	PICHINCHA	LARGE	RAINY	NEGATIVE	NEGATIVE	NEGATIVE	NEGATIVE	NEGATIVE	NEGATIVE
89	PICHINCHA	MEDIUM	RAINY	POSITIVE	NEGATIVE	NEGATIVE	NEGATIVE	POSITIVE	POSITIVE
90	MANABÍ	MEDIUM	RAINY	NEGATIVE	NEGATIVE	NEGATIVE	NEGATIVE	NEGATIVE	NEGATIVE
91	MANABÍ	MEDIUM	RAINY	NEGATIVE	NEGATIVE	NEGATIVE	NEGATIVE	NEGATIVE	NEGATIVE
92	MANABÍ	LARGE	RAINY	NEGATIVE	NEGATIVE	NEGATIVE	NEGATIVE	NEGATIVE	NEGATIVE
93	MANABÍ	SMALL	RAINY	NEGATIVE	NEGATIVE	NEGATIVE	NEGATIVE	NEGATIVE	NEGATIVE
94	MANABÍ	SMALL	RAINY	POSITIVE	NEGATIVE	POSITIVE	NEGATIVE	NEGATIVE	POSITIVE
95	MANABÍ	SMALL	RAINY	NEGATIVE	NEGATIVE	NEGATIVE	NEGATIVE	NEGATIVE	NEGATIVE
96	MANABÍ	LARGE	RAINY	NEGATIVE	NEGATIVE	NEGATIVE	NEGATIVE	NEGATIVE	NEGATIVE
97	MANABÍ	MEDIUM	RAINY	POSITIVE	NEGATIVE	POSITIVE	NEGATIVE	NEGATIVE	POSITIVE
98	MANABÍ	MEDIUM	RAINY	NEGATIVE	NEGATIVE	NEGATIVE	NEGATIVE	NEGATIVE	NEGATIVE
99	MANABÍ	LARGE	RAINY	NEGATIVE	NEGATIVE	NEGATIVE	NEGATIVE	NEGATIVE	NEGATIVE
100	MANABÍ	MEDIUM	RAINY	POSITIVE	POSITIVE	POSITIVE	POSITIVE	NEGATIVE	POSITIVE
101	MANABÍ	SMALL	RAINY	NEGATIVE	NEGATIVE	NEGATIVE	NEGATIVE	NEGATIVE	NEGATIVE
102	MANABÍ	MEDIUM	RAINY	NEGATIVE	NEGATIVE	NEGATIVE	NEGATIVE	NEGATIVE	NEGATIVE
103	MANABÍ	SMALL	RAINY	NEGATIVE	NEGATIVE	NEGATIVE	NEGATIVE	NEGATIVE	NEGATIVE
104	MANABÍ	SMALL	RAINY	POSITIVE	POSITIVE	NEGATIVE	NEGATIVE	NEGATIVE	POSITIVE
105	MANABÍ	LARGE	RAINY	POSITIVE	NEGATIVE	POSITIVE	NEGATIVE	NEGATIVE	POSITIVE
106	MANABÍ	SMALL	RAINY	POSITIVE	NEGATIVE	NEGATIVE	NEGATIVE	POSITIVE	POSITIVE
107	MANABÍ	MEDIUM	RAINY	NEGATIVE	NEGATIVE	NEGATIVE	NEGATIVE	NEGATIVE	NEGATIVE
108	MANABÍ	LARGE	RAINY	NEGATIVE	NEGATIVE	NEGATIVE	NEGATIVE	NEGATIVE	NEGATIVE
109	MANABÍ	SMALL	RAINY	NEGATIVE	NEGATIVE	NEGATIVE	NEGATIVE	NEGATIVE	NEGATIVE
110	PICHINCHA	SMALL	RAINY	NEGATIVE	NEGATIVE	NEGATIVE	NEGATIVE	NEGATIVE	NEGATIVE
111	PICHINCHA	SMALL	RAINY	NEGATIVE	NEGATIVE	NEGATIVE	NEGATIVE	NEGATIVE	NEGATIVE
112	PICHINCHA	SMALL	RAINY	NEGATIVE	NEGATIVE	NEGATIVE	NEGATIVE	NEGATIVE	NEGATIVE
113	PICHINCHA	SMALL	RAINY	POSITIVE	NEGATIVE	POSITIVE	NEGATIVE	NEGATIVE	POSITIVE
114	PICHINCHA	MEDIUM	RAINY	NEGATIVE	NEGATIVE	NEGATIVE	NEGATIVE	NEGATIVE	NEGATIVE
115	PICHINCHA	MEDIUM	RAINY	NEGATIVE	NEGATIVE	NEGATIVE	NEGATIVE	NEGATIVE	NEGATIVE
116	PICHINCHA	MEDIUM	RAINY	NEGATIVE	NEGATIVE	NEGATIVE	NEGATIVE	NEGATIVE	NEGATIVE
117	PICHINCHA	SMALL	RAINY	NEGATIVE	NEGATIVE	NEGATIVE	NEGATIVE	NEGATIVE	NEGATIVE

118	PICHINCHA	MEDIUM	RAINY	NEGATIVE	NEGATIVE	NEGATIVE	NEGATIVE	NEGATIVE	NEGATIVE
119	PICHINCHA	MEDIUM	RAINY	NEGATIVE	NEGATIVE	NEGATIVE	NEGATIVE	NEGATIVE	NEGATIVE
120	PICHINCHA	LARGE	RAINY	NEGATIVE	NEGATIVE	NEGATIVE	NEGATIVE	NEGATIVE	NEGATIVE
121	PICHINCHA	MEDIUM	RAINY	POSITIVE	NEGATIVE	POSITIVE	NEGATIVE	NEGATIVE	POSITIVE
122	PICHINCHA	LARGE	RAINY	POSITIVE	POSITIVE	POSITIVE	POSITIVE	NEGATIVE	POSITIVE
123	PICHINCHA	LARGE	RAINY	NEGATIVE	NEGATIVE	NEGATIVE	NEGATIVE	NEGATIVE	NEGATIVE
124	PICHINCHA	SMALL	RAINY	POSITIVE	POSITIVE	POSITIVE	POSITIVE	NEGATIVE	POSITIVE
125	PICHINCHA	MEDIUM	RAINY	NEGATIVE	NEGATIVE	NEGATIVE	NEGATIVE	NEGATIVE	NEGATIVE
126	PICHINCHA	LARGE	RAINY	NEGATIVE	NEGATIVE	NEGATIVE	NEGATIVE	NEGATIVE	NEGATIVE
127	PICHINCHA	LARGE	RAINY	NEGATIVE	NEGATIVE	NEGATIVE	NEGATIVE	NEGATIVE	NEGATIVE
128	PICHINCHA	SMALL	RAINY	NEGATIVE	NEGATIVE	NEGATIVE	NEGATIVE	NEGATIVE	NEGATIVE
129	PICHINCHA	SMALL	RAINY	NEGATIVE	NEGATIVE	NEGATIVE	NEGATIVE	NEGATIVE	NEGATIVE
130	PICHINCHA	LARGE	RAINY	NEGATIVE	NEGATIVE	NEGATIVE	NEGATIVE	NEGATIVE	NEGATIVE
131	PICHINCHA	MEDIUM	RAINY	POSITIVE	NEGATIVE	POSITIVE	NEGATIVE	NEGATIVE	POSITIVE
132	PICHINCHA	LARGE	RAINY	NEGATIVE	NEGATIVE	NEGATIVE	NEGATIVE	NEGATIVE	NEGATIVE
133	PICHINCHA	LARGE	RAINY	NEGATIVE	NEGATIVE	NEGATIVE	NEGATIVE	NEGATIVE	NEGATIVE
134	PICHINCHA	SMALL	RAINY	POSITIVE	POSITIVE	NEGATIVE	NEGATIVE	NEGATIVE	POSITIVE
135	PICHINCHA	LARGE	RAINY	POSITIVE	POSITIVE	POSITIVE	POSITIVE	NEGATIVE	POSITIVE
136	PICHINCHA	SMALL	RAINY	NEGATIVE	NEGATIVE	NEGATIVE	NEGATIVE	NEGATIVE	NEGATIVE
137	PICHINCHA	LARGE	RAINY	NEGATIVE	NEGATIVE	NEGATIVE	NEGATIVE	NEGATIVE	NEGATIVE
138	PICHINCHA	MEDIUM	RAINY	NEGATIVE	NEGATIVE	NEGATIVE	NEGATIVE	NEGATIVE	NEGATIVE
139	PICHINCHA	LARGE	RAINY	NEGATIVE	NEGATIVE	NEGATIVE	NEGATIVE	NEGATIVE	NEGATIVE
140	PICHINCHA	MEDIUM	RAINY	NEGATIVE	NEGATIVE	NEGATIVE	NEGATIVE	NEGATIVE	NEGATIVE
141	PICHINCHA	LARGE	RAINY	NEGATIVE	NEGATIVE	NEGATIVE	NEGATIVE	NEGATIVE	NEGATIVE
142	PICHINCHA	SMALL	RAINY	NEGATIVE	NEGATIVE	NEGATIVE	NEGATIVE	NEGATIVE	NEGATIVE
143	PICHINCHA	LARGE	RAINY	NEGATIVE	NEGATIVE	NEGATIVE	NEGATIVE	NEGATIVE	NEGATIVE
144	PICHINCHA	MEDIUM	RAINY	NEGATIVE	NEGATIVE	NEGATIVE	NEGATIVE	NEGATIVE	NEGATIVE
145	PICHINCHA	LARGE	RAINY	NEGATIVE	NEGATIVE	NEGATIVE	NEGATIVE	NEGATIVE	NEGATIVE
146	PICHINCHA	LARGE	RAINY	NEGATIVE	NEGATIVE	NEGATIVE	NEGATIVE	NEGATIVE	NEGATIVE
147	PICHINCHA	MEDIUM	RAINY	POSITIVE	POSITIVE	NEGATIVE	NEGATIVE	NEGATIVE	POSITIVE
148	PICHINCHA	SMALL	RAINY	POSITIVE	POSITIVE	NEGATIVE	NEGATIVE	NEGATIVE	POSITIVE

149	PICHINCHA	LARGE	RAINY	POSITIVE	POSITIVE	NEGATIVE	NEGATIVE	NEGATIVE	POSITIVE
150	PICHINCHA	LARGE	RAINY	POSITIVE	POSITIVE	POSITIVE	POSITIVE	NEGATIVE	POSITIVE
151	PICHINCHA	MEDIUM	RAINY	POSITIVE	NEGATIVE	NEGATIVE	NEGATIVE	POSITIVE	POSITIVE
152	PICHINCHA	SMALL	RAINY	POSITIVE	NEGATIVE	NEGATIVE	NEGATIVE	POSITIVE	POSITIVE
153	PICHINCHA	MEDIUM	RAINY	POSITIVE	NEGATIVE	POSITIVE	NEGATIVE	NEGATIVE	POSITIVE
154	PICHINCHA	LARGE	RAINY	NEGATIVE	NEGATIVE	NEGATIVE	NEGATIVE	NEGATIVE	NEGATIVE
155	PICHINCHA	LARGE	RAINY	POSITIVE	POSITIVE	POSITIVE	POSITIVE	NEGATIVE	POSITIVE
156	PICHINCHA	SMALL	RAINY	NEGATIVE	NEGATIVE	NEGATIVE	NEGATIVE	NEGATIVE	NEGATIVE
157	PICHINCHA	SMALL	RAINY	NEGATIVE	NEGATIVE	NEGATIVE	NEGATIVE	NEGATIVE	NEGATIVE
158	PICHINCHA	MEDIUM	RAINY	NEGATIVE	NEGATIVE	NEGATIVE	NEGATIVE	NEGATIVE	NEGATIVE
159	PICHINCHA	MEDIUM	RAINY	POSITIVE	NEGATIVE	NEGATIVE	NEGATIVE	POSITIVE	POSITIVE
160	PICHINCHA	MEDIUM	RAINY	NEGATIVE	NEGATIVE	NEGATIVE	NEGATIVE	NEGATIVE	NEGATIVE
161	PICHINCHA	SMALL	RAINY	NEGATIVE	NEGATIVE	NEGATIVE	NEGATIVE	NEGATIVE	NEGATIVE
162	PICHINCHA	MEDIUM	RAINY	NEGATIVE	NEGATIVE	NEGATIVE	NEGATIVE	NEGATIVE	NEGATIVE
163	PICHINCHA	MEDIUM	RAINY	NEGATIVE	NEGATIVE	NEGATIVE	NEGATIVE	NEGATIVE	NEGATIVE
164	PICHINCHA	LARGE	RAINY	NEGATIVE	NEGATIVE	NEGATIVE	NEGATIVE	NEGATIVE	NEGATIVE
165	PICHINCHA	MEDIUM	RAINY	POSITIVE	NEGATIVE	POSITIVE	NEGATIVE	NEGATIVE	POSITIVE
166	PICHINCHA	MEDIUM	RAINY	NEGATIVE	NEGATIVE	NEGATIVE	NEGATIVE	NEGATIVE	NEGATIVE
167	PICHINCHA	MEDIUM	RAINY	NEGATIVE	NEGATIVE	NEGATIVE	NEGATIVE	NEGATIVE	NEGATIVE
168	PICHINCHA	SMALL	RAINY	NEGATIVE	NEGATIVE	NEGATIVE	NEGATIVE	NEGATIVE	NEGATIVE
169	PICHINCHA	SMALL	RAINY	NEGATIVE	NEGATIVE	NEGATIVE	NEGATIVE	NEGATIVE	NEGATIVE
170	PICHINCHA	MEDIUM	RAINY	POSITIVE	NEGATIVE	NEGATIVE	NEGATIVE	POSITIVE	POSITIVE
171	PICHINCHA	MEDIUM	RAINY	NEGATIVE	NEGATIVE	NEGATIVE	NEGATIVE	NEGATIVE	NEGATIVE
172	PICHINCHA	SMALL	RAINY	POSITIVE	NEGATIVE	NEGATIVE	NEGATIVE	POSITIVE	POSITIVE
173	PICHINCHA	MEDIUM	RAINY	POSITIVE	POSITIVE	POSITIVE	POSITIVE	NEGATIVE	POSITIVE
174	PICHINCHA	LARGE	RAINY	NEGATIVE	NEGATIVE	NEGATIVE	NEGATIVE	NEGATIVE	NEGATIVE
175	PICHINCHA	SMALL	RAINY	NEGATIVE	NEGATIVE	NEGATIVE	NEGATIVE	NEGATIVE	NEGATIVE
176	PICHINCHA	MEDIUM	RAINY	NEGATIVE	NEGATIVE	NEGATIVE	NEGATIVE	NEGATIVE	NEGATIVE
177	PICHINCHA	SMALL	RAINY	NEGATIVE	NEGATIVE	NEGATIVE	NEGATIVE	NEGATIVE	NEGATIVE
178	PICHINCHA	LARGE	RAINY	NEGATIVE	NEGATIVE	NEGATIVE	NEGATIVE	NEGATIVE	NEGATIVE
179	PICHINCHA	MEDIUM	RAINY	POSITIVE	POSITIVE	POSITIVE	POSITIVE	NEGATIVE	POSITIVE

180	PICHINCHA	MEDIUM	RAINY	NEGATIVE	NEGATIVE	NEGATIVE	NEGATIVE	NEGATIVE	NEGATIVE
181	PICHINCHA	LARGE	RAINY	NEGATIVE	NEGATIVE	NEGATIVE	NEGATIVE	NEGATIVE	NEGATIVE
182	PICHINCHA	MEDIUM	RAINY	POSITIVE	POSITIVE	POSITIVE	POSITIVE	NEGATIVE	POSITIVE
183	PICHINCHA	MEDIUM	RAINY	NEGATIVE	NEGATIVE	NEGATIVE	NEGATIVE	NEGATIVE	NEGATIVE
184	PICHINCHA	LARGE	RAINY	NEGATIVE	NEGATIVE	NEGATIVE	NEGATIVE	NEGATIVE	NEGATIVE
185	PICHINCHA	MEDIUM	RAINY	NEGATIVE	NEGATIVE	NEGATIVE	NEGATIVE	NEGATIVE	NEGATIVE
186	PICHINCHA	LARGE	RAINY	NEGATIVE	NEGATIVE	NEGATIVE	NEGATIVE	NEGATIVE	NEGATIVE
187	PICHINCHA	LARGE	RAINY	NEGATIVE	NEGATIVE	NEGATIVE	NEGATIVE	NEGATIVE	NEGATIVE
188	PICHINCHA	LARGE	RAINY	NEGATIVE	NEGATIVE	NEGATIVE	NEGATIVE	NEGATIVE	NEGATIVE
189	PICHINCHA	LARGE	RAINY	NEGATIVE	NEGATIVE	NEGATIVE	NEGATIVE	NEGATIVE	NEGATIVE
190	PICHINCHA	MEDIUM	RAINY	NEGATIVE	NEGATIVE	NEGATIVE	NEGATIVE	NEGATIVE	NEGATIVE
191	PICHINCHA	LARGE	RAINY	NEGATIVE	NEGATIVE	NEGATIVE	NEGATIVE	NEGATIVE	NEGATIVE
192	PICHINCHA	MEDIUM	RAINY	POSITIVE	NEGATIVE	POSITIVE	NEGATIVE	NEGATIVE	POSITIVE
193	PICHINCHA	LARGE	RAINY	NEGATIVE	NEGATIVE	NEGATIVE	NEGATIVE	NEGATIVE	NEGATIVE
194	MANABÍ	LARGE	RAINY	POSITIVE	POSITIVE	POSITIVE	POSITIVE	NEGATIVE	POSITIVE
195	MANABÍ	MEDIUM	RAINY	NEGATIVE	NEGATIVE	NEGATIVE	NEGATIVE	NEGATIVE	NEGATIVE
196	MANABÍ	MEDIUM	RAINY	NEGATIVE	NEGATIVE	NEGATIVE	NEGATIVE	NEGATIVE	NEGATIVE
197	MANABÍ	MEDIUM	RAINY	POSITIVE	NEGATIVE	POSITIVE	NEGATIVE	NEGATIVE	POSITIVE
198	MANABÍ	SMALL	RAINY	POSITIVE	NEGATIVE	POSITIVE	NEGATIVE	NEGATIVE	POSITIVE
199	MANABÍ	LARGE	RAINY	NEGATIVE	NEGATIVE	NEGATIVE	NEGATIVE	NEGATIVE	NEGATIVE
200	MANABÍ	SMALL	RAINY	NEGATIVE	NEGATIVE	NEGATIVE	NEGATIVE	NEGATIVE	NEGATIVE
201	MANABÍ	MEDIUM	RAINY	POSITIVE	NEGATIVE	NEGATIVE	NEGATIVE	POSITIVE	POSITIVE
202	MANABÍ	MEDIUM	RAINY	POSITIVE	NEGATIVE	NEGATIVE	NEGATIVE	POSITIVE	POSITIVE
203	MANABÍ	SMALL	RAINY	POSITIVE	NEGATIVE	NEGATIVE	NEGATIVE	POSITIVE	POSITIVE
204	MANABÍ	LARGE	RAINY	POSITIVE	POSITIVE	NEGATIVE	NEGATIVE	NEGATIVE	POSITIVE
205	MANABÍ	LARGE	RAINY	NEGATIVE	NEGATIVE	NEGATIVE	NEGATIVE	NEGATIVE	NEGATIVE
206	MANABÍ	LARGE	RAINY	POSITIVE	POSITIVE	NEGATIVE	NEGATIVE	NEGATIVE	POSITIVE
207	MANABÍ	LARGE	RAINY	NEGATIVE	NEGATIVE	NEGATIVE	NEGATIVE	NEGATIVE	NEGATIVE
208	MANABÍ	MEDIUM	RAINY	NEGATIVE	NEGATIVE	NEGATIVE	NEGATIVE	NEGATIVE	NEGATIVE
209	MANABÍ	SMALL	RAINY	NEGATIVE	NEGATIVE	NEGATIVE	NEGATIVE	NEGATIVE	NEGATIVE
210	MANABÍ	MEDIUM	RAINY	POSITIVE	NEGATIVE	NEGATIVE	NEGATIVE	POSITIVE	POSITIVE

211	MANABÍ	LARGE	RAINY	POSITIVE	POSITIVE	NEGATIVE	NEGATIVE	NEGATIVE	POSITIVE
212	MANABÍ	SMALL	RAINY	NEGATIVE	NEGATIVE	NEGATIVE	NEGATIVE	NEGATIVE	NEGATIVE
213	MANABÍ	LARGE	RAINY	POSITIVE	POSITIVE	NEGATIVE	NEGATIVE	NEGATIVE	POSITIVE
214	MANABÍ	SMALL	RAINY	POSITIVE	NEGATIVE	POSITIVE	NEGATIVE	NEGATIVE	POSITIVE
215	MANABÍ	MEDIUM	RAINY	POSITIVE	POSITIVE	NEGATIVE	NEGATIVE	NEGATIVE	POSITIVE
216	MANABÍ	SMALL	RAINY	NEGATIVE	NEGATIVE	NEGATIVE	NEGATIVE	NEGATIVE	NEGATIVE
217	MANABÍ	LARGE	RAINY	POSITIVE	NEGATIVE	POSITIVE	NEGATIVE	NEGATIVE	POSITIVE
218	MANABÍ	SMALL	RAINY	POSITIVE	NEGATIVE	POSITIVE	NEGATIVE	NEGATIVE	POSITIVE
219	MANABÍ	SMALL	RAINY	NEGATIVE	NEGATIVE	NEGATIVE	NEGATIVE	NEGATIVE	NEGATIVE
220	MANABÍ	MEDIUM	RAINY	NEGATIVE	NEGATIVE	NEGATIVE	NEGATIVE	NEGATIVE	NEGATIVE
221	MANABÍ	SMALL	RAINY	NEGATIVE	NEGATIVE	NEGATIVE	NEGATIVE	NEGATIVE	NEGATIVE
222	MANABÍ	LARGE	RAINY	POSITIVE	NEGATIVE	NEGATIVE	NEGATIVE	POSITIVE	POSITIVE
223	MANABÍ	LARGE	RAINY	NEGATIVE	NEGATIVE	NEGATIVE	NEGATIVE	NEGATIVE	NEGATIVE
224	MANABÍ	LARGE	RAINY	POSITIVE	POSITIVE	POSITIVE	POSITIVE	NEGATIVE	POSITIVE
225	MANABÍ	SMALL	RAINY	NEGATIVE	NEGATIVE	NEGATIVE	NEGATIVE	NEGATIVE	NEGATIVE
226	MANABÍ	MEDIUM	RAINY	POSITIVE	NEGATIVE	POSITIVE	NEGATIVE	NEGATIVE	POSITIVE
227	MANABÍ	SMALL	RAINY	NEGATIVE	NEGATIVE	NEGATIVE	NEGATIVE	NEGATIVE	NEGATIVE
228	MANABÍ	MEDIUM	RAINY	POSITIVE	NEGATIVE	POSITIVE	NEGATIVE	NEGATIVE	POSITIVE
229	MANABÍ	SMALL	RAINY	POSITIVE	NEGATIVE	POSITIVE	NEGATIVE	NEGATIVE	POSITIVE
230	MANABÍ	MEDIUM	RAINY	POSITIVE	NEGATIVE	NEGATIVE	NEGATIVE	POSITIVE	POSITIVE
231	MANABÍ	SMALL	RAINY	NEGATIVE	NEGATIVE	NEGATIVE	NEGATIVE	NEGATIVE	NEGATIVE
232	MANABÍ	SMALL	RAINY	NEGATIVE	NEGATIVE	NEGATIVE	NEGATIVE	NEGATIVE	NEGATIVE
233	MANABÍ	MEDIUM	RAINY	NEGATIVE	NEGATIVE	NEGATIVE	NEGATIVE	NEGATIVE	NEGATIVE
234	MANABÍ	LARGE	WARM	POSITIVE	POSITIVE	NEGATIVE	NEGATIVE	NEGATIVE	POSITIVE
235	MANABÍ	MEDIUM	WARM	POSITIVE	NEGATIVE	NEGATIVE	NEGATIVE	POSITIVE	POSITIVE
236	MANABÍ	MEDIUM	WARM	POSITIVE	NEGATIVE	NEGATIVE	NEGATIVE	POSITIVE	POSITIVE
237	MANABÍ	LARGE	WARM	POSITIVE	NEGATIVE	NEGATIVE	NEGATIVE	POSITIVE	POSITIVE
238	MANABÍ	MEDIUM	WARM	POSITIVE	NEGATIVE	POSITIVE	NEGATIVE	NEGATIVE	POSITIVE
239	MANABÍ	LARGE	WARM	POSITIVE	NEGATIVE	NEGATIVE	NEGATIVE	POSITIVE	POSITIVE
240	MANABÍ	MEDIUM	WARM	POSITIVE	NEGATIVE	NEGATIVE	NEGATIVE	POSITIVE	POSITIVE
241	MANABÍ	SMALL	WARM	NEGATIVE	NEGATIVE	NEGATIVE	NEGATIVE	NEGATIVE	NEGATIVE

242	MANABÍ	MEDIUM	WARM	POSITIVE	POSITIVE	NEGATIVE	NEGATIVE	NEGATIVE	POSITIVE
243	MANABÍ	LARGE	WARM	POSITIVE	NEGATIVE	NEGATIVE	NEGATIVE	POSITIVE	POSITIVE
244	MANABÍ	SMALL	WARM	POSITIVE	POSITIVE	POSITIVE	POSITIVE	NEGATIVE	POSITIVE
245	MANABÍ	MEDIUM	WARM	POSITIVE	NEGATIVE	POSITIVE	NEGATIVE	NEGATIVE	POSITIVE
246	MANABÍ	SMALL	WARM	POSITIVE	NEGATIVE	NEGATIVE	NEGATIVE	POSITIVE	POSITIVE
247	MANABÍ	LARGE	WARM	POSITIVE	POSITIVE	POSITIVE	POSITIVE	NEGATIVE	POSITIVE
248	MANABÍ	MEDIUM	WARM	NEGATIVE	NEGATIVE	NEGATIVE	NEGATIVE	NEGATIVE	NEGATIVE
249	MANABÍ	SMALL	WARM	POSITIVE	NEGATIVE	POSITIVE	NEGATIVE	NEGATIVE	POSITIVE
250	MANABÍ	SMALL	WARM	NEGATIVE	NEGATIVE	NEGATIVE	NEGATIVE	NEGATIVE	NEGATIVE
251	MANABÍ	MEDIUM	WARM	NEGATIVE	NEGATIVE	NEGATIVE	NEGATIVE	NEGATIVE	NEGATIVE
252	MANABÍ	MEDIUM	WARM	POSITIVE	NEGATIVE	POSITIVE	NEGATIVE	NEGATIVE	POSITIVE
253	MANABÍ	LARGE	WARM	POSITIVE	NEGATIVE	NEGATIVE	NEGATIVE	POSITIVE	POSITIVE
254	MANABÍ	SMALL	WARM	NEGATIVE	NEGATIVE	NEGATIVE	NEGATIVE	NEGATIVE	NEGATIVE
255	MANABÍ	MEDIUM	WARM	NEGATIVE	NEGATIVE	NEGATIVE	NEGATIVE	NEGATIVE	NEGATIVE
256	MANABÍ	MEDIUM	WARM	POSITIVE	NEGATIVE	NEGATIVE	NEGATIVE	POSITIVE	POSITIVE
257	MANABÍ	LARGE	WARM	POSITIVE	NEGATIVE	NEGATIVE	NEGATIVE	POSITIVE	POSITIVE
258	MANABÍ	SMALL	WARM	NEGATIVE	NEGATIVE	NEGATIVE	NEGATIVE	NEGATIVE	NEGATIVE
259	MANABÍ	SMALL	WARM	POSITIVE	NEGATIVE	NEGATIVE	NEGATIVE	POSITIVE	POSITIVE
260	MANABÍ	MEDIUM	WARM	NEGATIVE	NEGATIVE	NEGATIVE	NEGATIVE	NEGATIVE	NEGATIVE
261	MANABÍ	MEDIUM	WARM	POSITIVE	POSITIVE	NEGATIVE	NEGATIVE	NEGATIVE	POSITIVE
262	MANABÍ	SMALL	WARM	POSITIVE	POSITIVE	POSITIVE	POSITIVE	NEGATIVE	POSITIVE
263	MANABÍ	LARGE	WARM	NEGATIVE	NEGATIVE	NEGATIVE	NEGATIVE	NEGATIVE	NEGATIVE
264	MANABÍ	LARGE	WARM	POSITIVE	NEGATIVE	NEGATIVE	NEGATIVE	POSITIVE	POSITIVE
265	MANABÍ	SMALL	WARM	POSITIVE	NEGATIVE	NEGATIVE	NEGATIVE	POSITIVE	POSITIVE
266	MANABÍ	MEDIUM	WARM	NEGATIVE	NEGATIVE	NEGATIVE	NEGATIVE	NEGATIVE	NEGATIVE
267	MANABÍ	LARGE	WARM	NEGATIVE	NEGATIVE	NEGATIVE	NEGATIVE	NEGATIVE	NEGATIVE
268	MANABÍ	SMALL	WARM	NEGATIVE	NEGATIVE	NEGATIVE	NEGATIVE	NEGATIVE	NEGATIVE
269	MANABÍ	MEDIUM	WARM	NEGATIVE	NEGATIVE	NEGATIVE	NEGATIVE	NEGATIVE	NEGATIVE
270	MANABÍ	SMALL	WARM	NEGATIVE	NEGATIVE	NEGATIVE	NEGATIVE	NEGATIVE	NEGATIVE
271	MANABÍ	MEDIUM	WARM	NEGATIVE	NEGATIVE	NEGATIVE	NEGATIVE	NEGATIVE	NEGATIVE
272	MANABÍ	SMALL	WARM	NEGATIVE	NEGATIVE	NEGATIVE	NEGATIVE	NEGATIVE	NEGATIVE

273	MANABÍ	MEDIUM	WARM	NEGATIVE	NEGATIVE	NEGATIVE	NEGATIVE	NEGATIVE	NEGATIVE
274	MANABÍ	LARGE	WARM	POSITIVE	NEGATIVE	NEGATIVE	NEGATIVE	POSITIVE	POSITIVE
275	MANABÍ	SMALL	WARM	POSITIVE	NEGATIVE	POSITIVE	NEGATIVE	NEGATIVE	POSITIVE
276	MANABÍ	SMALL	WARM	POSITIVE	NEGATIVE	NEGATIVE	NEGATIVE	POSITIVE	POSITIVE
277	MANABÍ	MEDIUM	WARM	POSITIVE	POSITIVE	NEGATIVE	NEGATIVE	NEGATIVE	POSITIVE
278	MANABÍ	LARGE	WARM	POSITIVE	NEGATIVE	POSITIVE	NEGATIVE	NEGATIVE	POSITIVE
279	MANABÍ	MEDIUM	WARM	POSITIVE	NEGATIVE	NEGATIVE	NEGATIVE	POSITIVE	POSITIVE
280	MANABÍ	SMALL	WARM	POSITIVE	NEGATIVE	NEGATIVE	NEGATIVE	POSITIVE	POSITIVE
281	MANABÍ	MEDIUM	WARM	POSITIVE	NEGATIVE	POSITIVE	NEGATIVE	NEGATIVE	POSITIVE
282	MANABÍ	SMALL	WARM	POSITIVE	NEGATIVE	POSITIVE	NEGATIVE	NEGATIVE	POSITIVE
283	MANABÍ	LARGE	WARM	POSITIVE	NEGATIVE	NEGATIVE	NEGATIVE	POSITIVE	POSITIVE
284	MANABÍ	SMALL	WARM	POSITIVE	NEGATIVE	POSITIVE	NEGATIVE	NEGATIVE	POSITIVE
285	MANABÍ	LARGE	WARM	POSITIVE	NEGATIVE	NEGATIVE	NEGATIVE	POSITIVE	POSITIVE
286	MANABÍ	LARGE	WARM	POSITIVE	NEGATIVE	POSITIVE	NEGATIVE	NEGATIVE	POSITIVE
287	MANABÍ	SMALL	WARM	POSITIVE	POSITIVE	POSITIVE	POSITIVE	NEGATIVE	POSITIVE
288	MANABÍ	MEDIUM	WARM	POSITIVE	NEGATIVE	POSITIVE	NEGATIVE	NEGATIVE	POSITIVE
289	MANABÍ	SMALL	WARM	POSITIVE	NEGATIVE	POSITIVE	NEGATIVE	NEGATIVE	POSITIVE
290	MANABÍ	LARGE	WARM	POSITIVE	POSITIVE	POSITIVE	POSITIVE	NEGATIVE	POSITIVE
291	MANABÍ	SMALL	WARM	POSITIVE	NEGATIVE	POSITIVE	NEGATIVE	NEGATIVE	POSITIVE
292	MANABÍ	MEDIUM	WARM	POSITIVE	NEGATIVE	POSITIVE	NEGATIVE	NEGATIVE	POSITIVE
293	MANABÍ	MEDIUM	WARM	POSITIVE	NEGATIVE	NEGATIVE	NEGATIVE	POSITIVE	POSITIVE
294	MANABÍ	SMALL	WARM	POSITIVE	NEGATIVE	NEGATIVE	NEGATIVE	POSITIVE	POSITIVE
295	MANABÍ	LARGE	WARM	POSITIVE	NEGATIVE	NEGATIVE	NEGATIVE	POSITIVE	POSITIVE
296	MANABÍ	SMALL	WARM	NEGATIVE	NEGATIVE	NEGATIVE	NEGATIVE	NEGATIVE	NEGATIVE
297	MANABÍ	MEDIUM	WARM	POSITIVE	NEGATIVE	NEGATIVE	NEGATIVE	POSITIVE	POSITIVE
298	MANABÍ	LARGE	WARM	POSITIVE	NEGATIVE	POSITIVE	NEGATIVE	NEGATIVE	POSITIVE
299	MANABÍ	LARGE	WARM	POSITIVE	NEGATIVE	POSITIVE	NEGATIVE	NEGATIVE	POSITIVE
300	MANABÍ	MEDIUM	WARM	POSITIVE	NEGATIVE	NEGATIVE	NEGATIVE	POSITIVE	POSITIVE
301	MANABÍ	SMALL	WARM	POSITIVE	NEGATIVE	NEGATIVE	NEGATIVE	POSITIVE	POSITIVE
302	MANABÍ	SMALL	WARM	NEGATIVE	NEGATIVE	NEGATIVE	NEGATIVE	NEGATIVE	NEGATIVE
303	MANABÍ	MEDIUM	WARM	NEGATIVE	NEGATIVE	NEGATIVE	NEGATIVE	NEGATIVE	NEGATIVE

304	MANABÍ	LARGE	WARM	POSITIVE	NEGATIVE	NEGATIVE	NEGATIVE	POSITIVE	NEGATIVE
305	MANABÍ	MEDIUM	WARM	POSITIVE	NEGATIVE	NEGATIVE	NEGATIVE	POSITIVE	POSITIVE
306	MANABÍ	SMALL	WARM	POSITIVE	NEGATIVE	NEGATIVE	NEGATIVE	POSITIVE	POSITIVE
307	MANABÍ	MEDIUM	WARM	POSITIVE	NEGATIVE	POSITIVE	NEGATIVE	NEGATIVE	POSITIVE
308	MANABÍ	SMALL	WARM	NEGATIVE	NEGATIVE	NEGATIVE	NEGATIVE	NEGATIVE	NEGATIVE
309	MANABÍ	LARGE	WARM	NEGATIVE	NEGATIVE	NEGATIVE	NEGATIVE	NEGATIVE	NEGATIVE
310	MANABÍ	SMALL	WARM	NEGATIVE	NEGATIVE	NEGATIVE	NEGATIVE	NEGATIVE	NEGATIVE
311	MANABÍ	LARGE	WARM	POSITIVE	NEGATIVE	NEGATIVE	NEGATIVE	POSITIVE	POSITIVE
312	MANABÍ	SMALL	WARM	POSITIVE	NEGATIVE	POSITIVE	NEGATIVE	NEGATIVE	POSITIVE
313	MANABÍ	LARGE	WARM	NEGATIVE	NEGATIVE	NEGATIVE	NEGATIVE	NEGATIVE	NEGATIVE
314	MANABÍ	SMALL	WARM	NEGATIVE	NEGATIVE	NEGATIVE	NEGATIVE	NEGATIVE	NEGATIVE
315	MANABÍ	MEDIUM	WARM	NEGATIVE	NEGATIVE	NEGATIVE	NEGATIVE	NEGATIVE	NEGATIVE
316	MANABÍ	MEDIUM	WARM	POSITIVE	NEGATIVE	NEGATIVE	NEGATIVE	POSITIVE	POSITIVE
317	MANABÍ	SMALL	WARM	NEGATIVE	NEGATIVE	NEGATIVE	NEGATIVE	NEGATIVE	NEGATIVE
318	MANABÍ	LARGE	WARM	POSITIVE	NEGATIVE	NEGATIVE	NEGATIVE	POSITIVE	POSITIVE
319	MANABÍ	SMALL	WARM	NEGATIVE	NEGATIVE	NEGATIVE	NEGATIVE	NEGATIVE	NEGATIVE
320	MANABÍ	LARGE	WARM	NEGATIVE	NEGATIVE	NEGATIVE	NEGATIVE	NEGATIVE	NEGATIVE
321	MANABÍ	SMALL	WARM	NEGATIVE	NEGATIVE	NEGATIVE	NEGATIVE	NEGATIVE	NEGATIVE
322	MANABÍ	LARGE	WARM	NEGATIVE	NEGATIVE	NEGATIVE	NEGATIVE	NEGATIVE	NEGATIVE
323	MANABÍ	LARGE	WARM	NEGATIVE	NEGATIVE	NEGATIVE	NEGATIVE	NEGATIVE	NEGATIVE
324	MANABÍ	SMALL	WARM	NEGATIVE	NEGATIVE	NEGATIVE	NEGATIVE	NEGATIVE	NEGATIVE
325	MANABÍ	MEDIUM	WARM	NEGATIVE	NEGATIVE	NEGATIVE	NEGATIVE	NEGATIVE	NEGATIVE
326	MANABÍ	LARGE	WARM	NEGATIVE	NEGATIVE	NEGATIVE	NEGATIVE	NEGATIVE	NEGATIVE
327	MANABÍ	MEDIUM	WARM	NEGATIVE	NEGATIVE	NEGATIVE	NEGATIVE	NEGATIVE	NEGATIVE
328	MANABÍ	SMALL	WARM	NEGATIVE	NEGATIVE	NEGATIVE	NEGATIVE	NEGATIVE	NEGATIVE
329	MANABÍ	MEDIUM	WARM	NEGATIVE	NEGATIVE	NEGATIVE	NEGATIVE	NEGATIVE	NEGATIVE
330	MANABÍ	SMALL	WARM	NEGATIVE	NEGATIVE	NEGATIVE	NEGATIVE	NEGATIVE	NEGATIVE
331	PICHINCHA	SMALL	WARM	NEGATIVE	NEGATIVE	NEGATIVE	NEGATIVE	NEGATIVE	NEGATIVE
332	PICHINCHA	SMALL	WARM	NEGATIVE	NEGATIVE	NEGATIVE	NEGATIVE	NEGATIVE	NEGATIVE
333	PICHINCHA	LARGE	WARM	POSITIVE	NEGATIVE	NEGATIVE	NEGATIVE	POSITIVE	POSITIVE
334	PICHINCHA	MEDIUM	WARM	NEGATIVE	NEGATIVE	NEGATIVE	NEGATIVE	NEGATIVE	NEGATIVE

335	PICHINCHA	SMALL	WARM	NEGATIVE	NEGATIVE	NEGATIVE	NEGATIVE	NEGATIVE	NEGATIVE
336	PICHINCHA	SMALL	WARM	NEGATIVE	NEGATIVE	NEGATIVE	NEGATIVE	NEGATIVE	NEGATIVE
337	PICHINCHA	SMALL	WARM	NEGATIVE	NEGATIVE	NEGATIVE	NEGATIVE	NEGATIVE	NEGATIVE
338	PICHINCHA	SMALL	WARM	POSITIVE	NEGATIVE	POSITIVE	NEGATIVE	NEGATIVE	POSITIVE
339	PICHINCHA	LARGE	WARM	POSITIVE	NEGATIVE	POSITIVE	NEGATIVE	NEGATIVE	NEGATIVE
340	PICHINCHA	MEDIUM	WARM	POSITIVE	NEGATIVE	NEGATIVE	NEGATIVE	POSITIVE	POSITIVE
341	PICHINCHA	SMALL	WARM	POSITIVE	NEGATIVE	POSITIVE	NEGATIVE	NEGATIVE	POSITIVE
342	PICHINCHA	SMALL	WARM	POSITIVE	POSITIVE	POSITIVE	POSITIVE	NEGATIVE	POSITIVE
343	PICHINCHA	SMALL	WARM	POSITIVE	POSITIVE	POSITIVE	POSITIVE	NEGATIVE	POSITIVE
344	PICHINCHA	SMALL	WARM	POSITIVE	NEGATIVE	POSITIVE	NEGATIVE	NEGATIVE	POSITIVE
345	PICHINCHA	MEDIUM	WARM	NEGATIVE	NEGATIVE	NEGATIVE	NEGATIVE	NEGATIVE	NEGATIVE
346	PICHINCHA	MEDIUM	WARM	POSITIVE	NEGATIVE	POSITIVE	NEGATIVE	NEGATIVE	POSITIVE
347	PICHINCHA	LARGE	WARM	POSITIVE	NEGATIVE	POSITIVE	NEGATIVE	NEGATIVE	NEGATIVE
348	PICHINCHA	SMALL	WARM	POSITIVE	NEGATIVE	POSITIVE	NEGATIVE	NEGATIVE	POSITIVE
349	PICHINCHA	MEDIUM	WARM	POSITIVE	POSITIVE	POSITIVE	POSITIVE	NEGATIVE	POSITIVE
350	PICHINCHA	MEDIUM	WARM	POSITIVE	POSITIVE	POSITIVE	POSITIVE	NEGATIVE	POSITIVE
351	PICHINCHA	LARGE	WARM	POSITIVE	NEGATIVE	NEGATIVE	NEGATIVE	POSITIVE	NEGATIVE
352	PICHINCHA	SMALL	WARM	POSITIVE	NEGATIVE	NEGATIVE	NEGATIVE	POSITIVE	POSITIVE
353	PICHINCHA	SMALL	WARM	POSITIVE	NEGATIVE	NEGATIVE	NEGATIVE	POSITIVE	POSITIVE
354	PICHINCHA	MEDIUM	WARM	POSITIVE	NEGATIVE	NEGATIVE	NEGATIVE	POSITIVE	POSITIVE
355	PICHINCHA	MEDIUM	WARM	POSITIVE	NEGATIVE	POSITIVE	NEGATIVE	NEGATIVE	POSITIVE
356	PICHINCHA	SMALL	WARM	POSITIVE	NEGATIVE	NEGATIVE	NEGATIVE	POSITIVE	POSITIVE
357	PICHINCHA	LARGE	WARM	NEGATIVE	NEGATIVE	NEGATIVE	NEGATIVE	NEGATIVE	NEGATIVE
358	PICHINCHA	SMALL	WARM	NEGATIVE	NEGATIVE	NEGATIVE	NEGATIVE	NEGATIVE	NEGATIVE
359	PICHINCHA	LARGE	WARM	POSITIVE	NEGATIVE	NEGATIVE	NEGATIVE	POSITIVE	POSITIVE
360	PICHINCHA	SMALL	WARM	NEGATIVE	NEGATIVE	NEGATIVE	NEGATIVE	NEGATIVE	NEGATIVE
361	PICHINCHA	LARGE	WARM	POSITIVE	NEGATIVE	NEGATIVE	NEGATIVE	POSITIVE	POSITIVE
362	PICHINCHA	LARGE	WARM	NEGATIVE	NEGATIVE	NEGATIVE	NEGATIVE	NEGATIVE	NEGATIVE
363	PICHINCHA	SMALL	WARM	NEGATIVE	NEGATIVE	NEGATIVE	NEGATIVE	NEGATIVE	NEGATIVE
364	PICHINCHA	MEDIUM	WARM	NEGATIVE	NEGATIVE	NEGATIVE	NEGATIVE	NEGATIVE	NEGATIVE
365	PICHINCHA	SMALL	WARM	NEGATIVE	NEGATIVE	NEGATIVE	NEGATIVE	NEGATIVE	NEGATIVE

366	PICHINCHA	LARGE	WARM	POSITIVE	NEGATIVE	NEGATIVE	NEGATIVE	POSITIVE	POSITIVE
367	PICHINCHA	SMALL	WARM	POSITIVE	NEGATIVE	POSITIVE	NEGATIVE	NEGATIVE	POSITIVE
368	PICHINCHA	SMALL	WARM	POSITIVE	NEGATIVE	NEGATIVE	NEGATIVE	POSITIVE	POSITIVE
369	PICHINCHA	LARGE	WARM	POSITIVE	NEGATIVE	POSITIVE	NEGATIVE	NEGATIVE	POSITIVE
370	PICHINCHA	SMALL	WARM	NEGATIVE	NEGATIVE	NEGATIVE	NEGATIVE	NEGATIVE	NEGATIVE
371	PICHINCHA	MEDIUM	WARM	NEGATIVE	NEGATIVE	NEGATIVE	NEGATIVE	NEGATIVE	NEGATIVE
372	PICHINCHA	SMALL	WARM	NEGATIVE	NEGATIVE	NEGATIVE	NEGATIVE	NEGATIVE	NEGATIVE
373	PICHINCHA	SMALL	WARM	POSITIVE	NEGATIVE	NEGATIVE	NEGATIVE	POSITIVE	POSITIVE
374	PICHINCHA	LARGE	WARM	NEGATIVE	NEGATIVE	NEGATIVE	NEGATIVE	NEGATIVE	NEGATIVE
375	PICHINCHA	MEDIUM	WARM	NEGATIVE	NEGATIVE	NEGATIVE	NEGATIVE	NEGATIVE	NEGATIVE
376	PICHINCHA	MEDIUM	WARM	NEGATIVE	NEGATIVE	NEGATIVE	NEGATIVE	NEGATIVE	NEGATIVE
377	PICHINCHA	SMALL	WARM	NEGATIVE	NEGATIVE	NEGATIVE	NEGATIVE	NEGATIVE	NEGATIVE
378	PICHINCHA	MEDIUM	WARM	POSITIVE	NEGATIVE	NEGATIVE	NEGATIVE	POSITIVE	POSITIVE
379	PICHINCHA	SMALL	WARM	NEGATIVE	NEGATIVE	NEGATIVE	NEGATIVE	NEGATIVE	NEGATIVE
380	PICHINCHA	SMALL	WARM	NEGATIVE	NEGATIVE	NEGATIVE	NEGATIVE	NEGATIVE	NEGATIVE
381	PICHINCHA	LARGE	WARM	POSITIVE	NEGATIVE	POSITIVE	NEGATIVE	NEGATIVE	POSITIVE
382	PICHINCHA	SMALL	WARM	POSITIVE	NEGATIVE	NEGATIVE	NEGATIVE	POSITIVE	POSITIVE
383	PICHINCHA	MEDIUM	WARM	POSITIVE	POSITIVE	NEGATIVE	NEGATIVE	NEGATIVE	POSITIVE
384	PICHINCHA	LARGE	WARM	NEGATIVE	NEGATIVE	NEGATIVE	NEGATIVE	NEGATIVE	NEGATIVE
385	PICHINCHA	SMALL	WARM	NEGATIVE	NEGATIVE	NEGATIVE	NEGATIVE	NEGATIVE	NEGATIVE
386	PICHINCHA	LARGE	WARM	POSITIVE	NEGATIVE	NEGATIVE	NEGATIVE	POSITIVE	POSITIVE
387	PICHINCHA	SMALL	WARM	NEGATIVE	NEGATIVE	NEGATIVE	NEGATIVE	NEGATIVE	NEGATIVE
388	PICHINCHA	LARGE	WARM	POSITIVE	NEGATIVE	POSITIVE	NEGATIVE	NEGATIVE	POSITIVE
389	PICHINCHA	SMALL	WARM	POSITIVE	NEGATIVE	POSITIVE	NEGATIVE	NEGATIVE	POSITIVE
390	PICHINCHA	LARGE	WARM	NEGATIVE	NEGATIVE	NEGATIVE	NEGATIVE	NEGATIVE	NEGATIVE
391	PICHINCHA	SMALL	WARM	POSITIVE	NEGATIVE	NEGATIVE	NEGATIVE	POSITIVE	POSITIVE
392	PICHINCHA	LARGE	WARM	NEGATIVE	NEGATIVE	NEGATIVE	NEGATIVE	NEGATIVE	NEGATIVE
393	PICHINCHA	LARGE	WARM	NEGATIVE	NEGATIVE	NEGATIVE	NEGATIVE	NEGATIVE	NEGATIVE
394	PICHINCHA	SMALL	WARM	NEGATIVE	NEGATIVE	NEGATIVE	NEGATIVE	NEGATIVE	NEGATIVE
395	PICHINCHA	MEDIUM	WARM	POSITIVE	NEGATIVE	POSITIVE	NEGATIVE	NEGATIVE	POSITIVE
396	MANABÍ	MEDIUM	WARM	POSITIVE	NEGATIVE	NEGATIVE	NEGATIVE	POSITIVE	POSITIVE

397	MANABÍ	LARGE	WARM	POSITIVE	NEGATIVE	POSITIVE	NEGATIVE	NEGATIVE	POSITIVE
398	MANABÍ	MEDIUM	WARM	POSITIVE	NEGATIVE	NEGATIVE	NEGATIVE	POSITIVE	POSITIVE
399	MANABÍ	SMALL	WARM	POSITIVE	NEGATIVE	NEGATIVE	NEGATIVE	POSITIVE	POSITIVE
400	MANABÍ	SMALL	WARM	NEGATIVE	NEGATIVE	NEGATIVE	NEGATIVE	NEGATIVE	NEGATIVE
401	MANABÍ	LARGE	WARM	POSITIVE	NEGATIVE	POSITIVE	NEGATIVE	NEGATIVE	POSITIVE
402	MANABÍ	SMALL	WARM	POSITIVE	NEGATIVE	POSITIVE	NEGATIVE	NEGATIVE	POSITIVE
403	MANABÍ	LARGE	WARM	POSITIVE	NEGATIVE	NEGATIVE	NEGATIVE	POSITIVE	POSITIVE
404	MANABÍ	SMALL	WARM	POSITIVE	POSITIVE	NEGATIVE	NEGATIVE	NEGATIVE	POSITIVE
405	MANABÍ	MEDIUM	WARM	NEGATIVE	NEGATIVE	NEGATIVE	NEGATIVE	NEGATIVE	NEGATIVE
406	MANABÍ	SMALL	WARM	NEGATIVE	NEGATIVE	NEGATIVE	NEGATIVE	NEGATIVE	NEGATIVE
407	MANABÍ	LARGE	WARM	NEGATIVE	NEGATIVE	NEGATIVE	NEGATIVE	NEGATIVE	NEGATIVE
408	MANABÍ	LARGE	WARM	POSITIVE	NEGATIVE	NEGATIVE	NEGATIVE	POSITIVE	NEGATIVE
409	MANABÍ	SMALL	WARM	POSITIVE	NEGATIVE	POSITIVE	NEGATIVE	NEGATIVE	POSITIVE
410	MANABÍ	LARGE	WARM	NEGATIVE	NEGATIVE	NEGATIVE	NEGATIVE	NEGATIVE	NEGATIVE
411	MANABÍ	LARGE	WARM	POSITIVE	NEGATIVE	POSITIVE	NEGATIVE	NEGATIVE	POSITIVE
412	MANABÍ	LARGE	WARM	POSITIVE	POSITIVE	NEGATIVE	NEGATIVE	NEGATIVE	POSITIVE
413	MANABÍ	SMALL	WARM	POSITIVE	NEGATIVE	NEGATIVE	NEGATIVE	POSITIVE	POSITIVE
414	MANABÍ	SMALL	WARM	POSITIVE	NEGATIVE	NEGATIVE	NEGATIVE	POSITIVE	POSITIVE
415	MANABÍ	MEDIUM	WARM	POSITIVE	NEGATIVE	NEGATIVE	NEGATIVE	POSITIVE	POSITIVE
416	MANABÍ	SMALL	WARM	POSITIVE	NEGATIVE	NEGATIVE	NEGATIVE	POSITIVE	POSITIVE
417	MANABÍ	LARGE	WARM	POSITIVE	NEGATIVE	NEGATIVE	NEGATIVE	POSITIVE	POSITIVE
418	MANABÍ	SMALL	WARM	POSITIVE	NEGATIVE	NEGATIVE	NEGATIVE	POSITIVE	POSITIVE
419	MANABÍ	SMALL	WARM	POSITIVE	POSITIVE	NEGATIVE	NEGATIVE	NEGATIVE	POSITIVE
420	MANABÍ	LARGE	WARM	POSITIVE	POSITIVE	NEGATIVE	NEGATIVE	NEGATIVE	POSITIVE
421	MANABÍ	MEDIUM	WARM	POSITIVE	POSITIVE	NEGATIVE	NEGATIVE	NEGATIVE	NEGATIVE
422	MANABÍ	MEDIUM	WARM	POSITIVE	POSITIVE	NEGATIVE	NEGATIVE	NEGATIVE	POSITIVE
423	MANABÍ	SMALL	WARM	POSITIVE	NEGATIVE	NEGATIVE	NEGATIVE	POSITIVE	POSITIVE
424	MANABÍ	LARGE	WARM	POSITIVE	NEGATIVE	NEGATIVE	NEGATIVE	POSITIVE	NEGATIVE
425	MANABÍ	SMALL	WARM	POSITIVE	NEGATIVE	NEGATIVE	NEGATIVE	POSITIVE	POSITIVE
426	MANABÍ	SMALL	WARM	NEGATIVE	NEGATIVE	NEGATIVE	NEGATIVE	NEGATIVE	NEGATIVE
427	MANABÍ	MEDIUM	WARM	NEGATIVE	NEGATIVE	NEGATIVE	NEGATIVE	NEGATIVE	NEGATIVE

428	MANABÍ	MEDIUM	WARM	NEGATIVE	NEGATIVE	NEGATIVE	NEGATIVE	NEGATIVE	NEGATIVE
429	MANABÍ	SMALL	WARM	NEGATIVE	NEGATIVE	NEGATIVE	NEGATIVE	NEGATIVE	NEGATIVE
430	MANABÍ	SMALL	WARM	NEGATIVE	NEGATIVE	NEGATIVE	NEGATIVE	NEGATIVE	NEGATIVE
431	MANABÍ	LARGE	WARM	NEGATIVE	NEGATIVE	NEGATIVE	NEGATIVE	NEGATIVE	NEGATIVE
432	MANABÍ	LARGE	WARM	NEGATIVE	NEGATIVE	NEGATIVE	NEGATIVE	NEGATIVE	NEGATIVE
433	MANABÍ	SMALL	WARM	NEGATIVE	NEGATIVE	NEGATIVE	NEGATIVE	NEGATIVE	NEGATIVE
434	MANABÍ	MEDIUM	WARM	NEGATIVE	NEGATIVE	NEGATIVE	NEGATIVE	NEGATIVE	NEGATIVE
435	MANABÍ	SMALL	WARM	NEGATIVE	NEGATIVE	NEGATIVE	NEGATIVE	NEGATIVE	NEGATIVE
436	MANABÍ	MEDIUM	WARM	NEGATIVE	NEGATIVE	NEGATIVE	NEGATIVE	NEGATIVE	NEGATIVE
437	MANABÍ	SMALL	WARM	NEGATIVE	NEGATIVE	NEGATIVE	NEGATIVE	NEGATIVE	NEGATIVE
438	MANABÍ	LARGE	WARM	NEGATIVE	NEGATIVE	NEGATIVE	NEGATIVE	NEGATIVE	NEGATIVE
439	MANABÍ	LARGE	WARM	NEGATIVE	NEGATIVE	NEGATIVE	NEGATIVE	NEGATIVE	NEGATIVE
440	MANABÍ	SMALL	WARM	NEGATIVE	NEGATIVE	NEGATIVE	NEGATIVE	NEGATIVE	NEGATIVE
441	MANABÍ	MEDIUM	WARM	NEGATIVE	NEGATIVE	NEGATIVE	NEGATIVE	NEGATIVE	NEGATIVE
442	MANABÍ	MEDIUM	WARM	NEGATIVE	NEGATIVE	NEGATIVE	NEGATIVE	NEGATIVE	NEGATIVE
443	MANABÍ	LARGE	WARM	NEGATIVE	NEGATIVE	NEGATIVE	NEGATIVE	NEGATIVE	NEGATIVE
444	MANABÍ	SMALL	WARM	NEGATIVE	NEGATIVE	NEGATIVE	NEGATIVE	NEGATIVE	NEGATIVE
445	MANABÍ	LARGE	WARM	POSITIVE	NEGATIVE	POSITIVE	NEGATIVE	NEGATIVE	POSITIVE
446	MANABÍ	SMALL	WARM	NEGATIVE	NEGATIVE	NEGATIVE	NEGATIVE	NEGATIVE	NEGATIVE
447	MANABÍ	MEDIUM	WARM	POSITIVE	POSITIVE	POSITIVE	POSITIVE	NEGATIVE	POSITIVE
448	MANABÍ	LARGE	WARM	POSITIVE	POSITIVE	POSITIVE	POSITIVE	NEGATIVE	POSITIVE
449	MANABÍ	SMALL	WARM	NEGATIVE	NEGATIVE	NEGATIVE	NEGATIVE	NEGATIVE	NEGATIVE
450	MANABÍ	SMALL	WARM	POSITIVE	NEGATIVE	NEGATIVE	NEGATIVE	POSITIVE	POSITIVE
451	MANABÍ	LARGE	WARM	POSITIVE	NEGATIVE	NEGATIVE	NEGATIVE	POSITIVE	POSITIVE
452	PICHINCHA	SMALL	WARM	POSITIVE	NEGATIVE	NEGATIVE	NEGATIVE	POSITIVE	POSITIVE
453	PICHINCHA	SMALL	WARM	NEGATIVE	NEGATIVE	NEGATIVE	NEGATIVE	NEGATIVE	NEGATIVE
454	PICHINCHA	MEDIUM	WARM	NEGATIVE	NEGATIVE	NEGATIVE	NEGATIVE	NEGATIVE	NEGATIVE
455	PICHINCHA	SMALL	WARM	NEGATIVE	NEGATIVE	NEGATIVE	NEGATIVE	NEGATIVE	NEGATIVE
456	MANABÍ	SMALL	WARM	NEGATIVE	NEGATIVE	NEGATIVE	NEGATIVE	NEGATIVE	NEGATIVE
457	MANABÍ	MEDIUM	WARM	NEGATIVE	NEGATIVE	NEGATIVE	NEGATIVE	NEGATIVE	NEGATIVE
458	MANABÍ	LARGE	WARM	NEGATIVE	NEGATIVE	NEGATIVE	NEGATIVE	NEGATIVE	NEGATIVE

459	MANABÍ	MEDIUM	WARM	POSITIVE	NEGATIVE	NEGATIVE	NEGATIVE	POSITIVE	POSITIVE
460	MANABÍ	SMALL	WARM	NEGATIVE	NEGATIVE	NEGATIVE	NEGATIVE	NEGATIVE	NEGATIVE
461	MANABÍ	SMALL	WARM	NEGATIVE	NEGATIVE	NEGATIVE	NEGATIVE	NEGATIVE	NEGATIVE
462	MANABÍ	LARGE	WARM	NEGATIVE	NEGATIVE	NEGATIVE	NEGATIVE	NEGATIVE	NEGATIVE
463	MANABÍ	SMALL	WARM	POSITIVE	POSITIVE	POSITIVE	POSITIVE	NEGATIVE	NEGATIVE
464	MANABÍ	LARGE	WARM	NEGATIVE	NEGATIVE	NEGATIVE	NEGATIVE	NEGATIVE	NEGATIVE
465	MANABÍ	SMALL	WARM	NEGATIVE	NEGATIVE	NEGATIVE	NEGATIVE	NEGATIVE	NEGATIVE
466	MANABÍ	MEDIUM	WARM	NEGATIVE	NEGATIVE	NEGATIVE	NEGATIVE	NEGATIVE	NEGATIVE
467	MANABÍ	SMALL	WARM	NEGATIVE	NEGATIVE	NEGATIVE	NEGATIVE	NEGATIVE	NEGATIVE
468	MANABÍ	LARGE	WARM	NEGATIVE	NEGATIVE	NEGATIVE	NEGATIVE	NEGATIVE	NEGATIVE
469	MANABÍ	LARGE	WARM	NEGATIVE	NEGATIVE	NEGATIVE	NEGATIVE	NEGATIVE	NEGATIVE
470	MANABÍ	SMALL	WARM	NEGATIVE	NEGATIVE	NEGATIVE	NEGATIVE	NEGATIVE	NEGATIVE
471	MANABÍ	LARGE	WARM	POSITIVE	NEGATIVE	NEGATIVE	NEGATIVE	POSITIVE	POSITIVE
472	MANABÍ	LARGE	WARM	NEGATIVE	NEGATIVE	NEGATIVE	NEGATIVE	NEGATIVE	NEGATIVE
473	MANABÍ	LARGE	WARM	NEGATIVE	NEGATIVE	NEGATIVE	NEGATIVE	NEGATIVE	NEGATIVE
474	MANABÍ	SMALL	WARM	POSITIVE	NEGATIVE	NEGATIVE	NEGATIVE	POSITIVE	POSITIVE
475	MANABÍ	SMALL	WARM	POSITIVE	NEGATIVE	POSITIVE	NEGATIVE	NEGATIVE	POSITIVE
476	MANABÍ	MEDIUM	WARM	NEGATIVE	NEGATIVE	NEGATIVE	NEGATIVE	NEGATIVE	NEGATIVE
477	MANABÍ	SMALL	WARM	NEGATIVE	NEGATIVE	NEGATIVE	NEGATIVE	NEGATIVE	NEGATIVE
478	MANABÍ	LARGE	WARM	NEGATIVE	NEGATIVE	NEGATIVE	NEGATIVE	NEGATIVE	NEGATIVE
479	MANABÍ	SMALL	WARM	POSITIVE	NEGATIVE	NEGATIVE	NEGATIVE	POSITIVE	POSITIVE
480	MANABÍ	SMALL	WARM	POSITIVE	NEGATIVE	NEGATIVE	NEGATIVE	POSITIVE	NEGATIVE
481	MANABÍ	LARGE	WARM	NEGATIVE	NEGATIVE	NEGATIVE	NEGATIVE	NEGATIVE	NEGATIVE
482	MANABÍ	MEDIUM	WARM	POSITIVE	NEGATIVE	POSITIVE	NEGATIVE	NEGATIVE	POSITIVE
483	MANABÍ	LARGE	WARM	POSITIVE	NEGATIVE	NEGATIVE	NEGATIVE	POSITIVE	POSITIVE
484	PICHINCHA	MEDIUM	WARM	POSITIVE	NEGATIVE	POSITIVE	NEGATIVE	NEGATIVE	POSITIVE
485	PICHINCHA	MEDIUM	WARM	POSITIVE	NEGATIVE	NEGATIVE	NEGATIVE	POSITIVE	NEGATIVE
486	PICHINCHA	SMALL	WARM	POSITIVE	NEGATIVE	NEGATIVE	NEGATIVE	POSITIVE	POSITIVE
487	PICHINCHA	LARGE	WARM	POSITIVE	NEGATIVE	POSITIVE	NEGATIVE	NEGATIVE	POSITIVE
488	PICHINCHA	SMALL	WARM	POSITIVE	NEGATIVE	POSITIVE	NEGATIVE	NEGATIVE	POSITIVE
489	PICHINCHA	SMALL	WARM	POSITIVE	NEGATIVE	NEGATIVE	NEGATIVE	POSITIVE	NEGATIVE

490	PICHINCHA	MEDIUM	WARM	NEGATIVE	NEGATIVE	NEGATIVE	NEGATIVE	NEGATIVE	NEGATIVE
491	PICHINCHA	MEDIUM	WARM	NEGATIVE	NEGATIVE	NEGATIVE	NEGATIVE	NEGATIVE	NEGATIVE
492	PICHINCHA	SMALL	WARM	NEGATIVE	NEGATIVE	NEGATIVE	NEGATIVE	NEGATIVE	NEGATIVE
493	PICHINCHA	SMALL	WARM	POSITIVE	NEGATIVE	POSITIVE	NEGATIVE	NEGATIVE	POSITIVE
494	PICHINCHA	LARGE	WARM	NEGATIVE	NEGATIVE	NEGATIVE	NEGATIVE	NEGATIVE	NEGATIVE
495	PICHINCHA	LARGE	WARM	POSITIVE	NEGATIVE	NEGATIVE	NEGATIVE	POSITIVE	POSITIVE
496	PICHINCHA	SMALL	WARM	NEGATIVE	NEGATIVE	NEGATIVE	NEGATIVE	NEGATIVE	NEGATIVE
497	PICHINCHA	MEDIUM	WARM	POSITIVE	NEGATIVE	POSITIVE	NEGATIVE	NEGATIVE	POSITIVE
498	PICHINCHA	SMALL	WARM	POSITIVE	NEGATIVE	POSITIVE	NEGATIVE	NEGATIVE	POSITIVE
499	PICHINCHA	MEDIUM	WARM	POSITIVE	POSITIVE	POSITIVE	POSITIVE	NEGATIVE	POSITIVE
500	PICHINCHA	SMALL	WARM	POSITIVE	NEGATIVE	NEGATIVE	NEGATIVE	POSITIVE	POSITIVE
501	PICHINCHA	LARGE	WARM	POSITIVE	NEGATIVE	POSITIVE	NEGATIVE	NEGATIVE	POSITIVE
502	PICHINCHA	LARGE	WARM	NEGATIVE	NEGATIVE	NEGATIVE	NEGATIVE	NEGATIVE	NEGATIVE
503	PICHINCHA	SMALL	WARM	POSITIVE	POSITIVE	POSITIVE	POSITIVE	NEGATIVE	POSITIVE
504	PICHINCHA	SMALL	WARM	NEGATIVE	NEGATIVE	NEGATIVE	NEGATIVE	NEGATIVE	NEGATIVE
505	PICHINCHA	SMALL	WARM	POSITIVE	NEGATIVE	POSITIVE	NEGATIVE	NEGATIVE	POSITIVE
506	PICHINCHA	LARGE	WARM	POSITIVE	POSITIVE	POSITIVE	POSITIVE	NEGATIVE	POSITIVE
507	PICHINCHA	SMALL	WARM	POSITIVE	NEGATIVE	NEGATIVE	NEGATIVE	POSITIVE	POSITIVE
508	PICHINCHA	MEDIUM	WARM	NEGATIVE	NEGATIVE	NEGATIVE	NEGATIVE	NEGATIVE	NEGATIVE
509	PICHINCHA	SMALL	WARM	POSITIVE	NEGATIVE	NEGATIVE	NEGATIVE	POSITIVE	NEGATIVE
510	PICHINCHA	LARGE	WARM	POSITIVE	NEGATIVE	POSITIVE	NEGATIVE	NEGATIVE	POSITIVE
511	PICHINCHA	MEDIUM	WARM	POSITIVE	POSITIVE	POSITIVE	POSITIVE	NEGATIVE	NEGATIVE
512	PICHINCHA	SMALL	WARM	POSITIVE	POSITIVE	POSITIVE	POSITIVE	NEGATIVE	POSITIVE
513	PICHINCHA	LARGE	WARM	NEGATIVE	NEGATIVE	NEGATIVE	NEGATIVE	NEGATIVE	NEGATIVE
514	PICHINCHA	SMALL	WARM	POSITIVE	NEGATIVE	POSITIVE	NEGATIVE	NEGATIVE	NEGATIVE
515	PICHINCHA	SMALL	WARM	POSITIVE	NEGATIVE	POSITIVE	NEGATIVE	NEGATIVE	POSITIVE
516	MANABÍ	MEDIUM	WARM	POSITIVE	NEGATIVE	POSITIVE	NEGATIVE	NEGATIVE	POSITIVE
517	MANABÍ	MEDIUM	WARM	POSITIVE	NEGATIVE	POSITIVE	NEGATIVE	NEGATIVE	POSITIVE
518	MANABÍ	LARGE	WARM	POSITIVE	POSITIVE	POSITIVE	POSITIVE	NEGATIVE	NEGATIVE
519	MANABÍ	SMALL	WARM	POSITIVE	NEGATIVE	NEGATIVE	NEGATIVE	POSITIVE	POSITIVE
520	MANABÍ	SMALL	WARM	POSITIVE	NEGATIVE	NEGATIVE	NEGATIVE	POSITIVE	POSITIVE

521	MANABÍ	SMALL	WARM	POSITIVE	POSITIVE	NEGATIVE	NEGATIVE	NEGATIVE	POSITIVE
522	MANABÍ	LARGE	WARM	POSITIVE	POSITIVE	NEGATIVE	NEGATIVE	NEGATIVE	POSITIVE
523	MANABÍ	MEDIUM	WARM	NEGATIVE	NEGATIVE	NEGATIVE	NEGATIVE	NEGATIVE	NEGATIVE
524	MANABÍ	MEDIUM	WARM	NEGATIVE	NEGATIVE	NEGATIVE	NEGATIVE	NEGATIVE	NEGATIVE
525	MANABÍ	LARGE	WARM	POSITIVE	NEGATIVE	NEGATIVE	NEGATIVE	POSITIVE	POSITIVE
526	MANABÍ	MEDIUM	WARM	POSITIVE	NEGATIVE	NEGATIVE	NEGATIVE	POSITIVE	POSITIVE
527	MANABÍ	SMALL	WARM	POSITIVE	NEGATIVE	POSITIVE	NEGATIVE	NEGATIVE	NEGATIVE
528	MANABÍ	MEDIUM	WARM	NEGATIVE	NEGATIVE	NEGATIVE	NEGATIVE	NEGATIVE	NEGATIVE
529	MANABÍ	SMALL	WARM	POSITIVE	NEGATIVE	NEGATIVE	NEGATIVE	POSITIVE	POSITIVE
530	MANABÍ	SMALL	WARM	NEGATIVE	NEGATIVE	NEGATIVE	NEGATIVE	NEGATIVE	NEGATIVE
531	MANABÍ	LARGE	WARM	POSITIVE	NEGATIVE	NEGATIVE	NEGATIVE	POSITIVE	NEGATIVE
532	MANABÍ	SMALL	WARM	NEGATIVE	NEGATIVE	NEGATIVE	NEGATIVE	NEGATIVE	NEGATIVE
533	MANABÍ	MEDIUM	WARM	NEGATIVE	NEGATIVE	NEGATIVE	NEGATIVE	NEGATIVE	NEGATIVE
534	MANABÍ	LARGE	WARM	POSITIVE	NEGATIVE	NEGATIVE	NEGATIVE	POSITIVE	POSITIVE
535	MANABÍ	SMALL	WARM	POSITIVE	NEGATIVE	POSITIVE	NEGATIVE	NEGATIVE	POSITIVE
536	MANABÍ	SMALL	WARM	POSITIVE	NEGATIVE	POSITIVE	NEGATIVE	NEGATIVE	POSITIVE
537	MANABÍ	SMALL	WARM	POSITIVE	NEGATIVE	NEGATIVE	NEGATIVE	POSITIVE	POSITIVE
538	MANABÍ	SMALL	WARM	POSITIVE	NEGATIVE	NEGATIVE	NEGATIVE	POSITIVE	POSITIVE
539	MANABÍ	SMALL	WARM	POSITIVE	NEGATIVE	POSITIVE	NEGATIVE	NEGATIVE	POSITIVE
540	MANABÍ	MEDIUM	WARM	POSITIVE	POSITIVE	POSITIVE	POSITIVE	NEGATIVE	POSITIVE
541	MANABÍ	MEDIUM	WARM	POSITIVE	NEGATIVE	NEGATIVE	NEGATIVE	POSITIVE	POSITIVE
542	MANABÍ	MEDIUM	WARM	POSITIVE	NEGATIVE	NEGATIVE	NEGATIVE	POSITIVE	POSITIVE
543	MANABÍ	LARGE	WARM	POSITIVE	NEGATIVE	NEGATIVE	NEGATIVE	POSITIVE	POSITIVE
544	MANABÍ	LARGE	WARM	POSITIVE	NEGATIVE	NEGATIVE	NEGATIVE	POSITIVE	POSITIVE
545	MANABÍ	MEDIUM	WARM	POSITIVE	NEGATIVE	POSITIVE	NEGATIVE	NEGATIVE	POSITIVE
546	MANABÍ	SMALL	WARM	POSITIVE	NEGATIVE	NEGATIVE	NEGATIVE	POSITIVE	POSITIVE
547	MANABÍ	MEDIUM	WARM	POSITIVE	NEGATIVE	NEGATIVE	NEGATIVE	POSITIVE	POSITIVE
548	MANABÍ	LARGE	WARM	POSITIVE	NEGATIVE	NEGATIVE	NEGATIVE	POSITIVE	NEGATIVE
549	MANABÍ	LARGE	WARM	POSITIVE	NEGATIVE	NEGATIVE	NEGATIVE	POSITIVE	POSITIVE
550	MANABÍ	SMALL	WARM	POSITIVE	POSITIVE	NEGATIVE	NEGATIVE	NEGATIVE	POSITIVE
551	MANABÍ	SMALL	WARM	POSITIVE	POSITIVE	NEGATIVE	NEGATIVE	NEGATIVE	POSITIVE

552	MANABÍ	MEDIUM	WARM	POSITIVE	NEGATIVE	NEGATIVE	NEGATIVE	POSITIVE	POSITIVE
553	MANABÍ	MEDIUM	WARM	POSITIVE	NEGATIVE	NEGATIVE	NEGATIVE	POSITIVE	POSITIVE
554	MANABÍ	LARGE	WARM	POSITIVE	NEGATIVE	NEGATIVE	NEGATIVE	POSITIVE	POSITIVE
555	MANABÍ	SMALL	WARM	POSITIVE	NEGATIVE	POSITIVE	NEGATIVE	NEGATIVE	POSITIVE
556	MANABÍ	MEDIUM	WARM	POSITIVE	NEGATIVE	POSITIVE	NEGATIVE	NEGATIVE	POSITIVE
557	MANABÍ	MEDIUM	WARM	POSITIVE	NEGATIVE	NEGATIVE	NEGATIVE	POSITIVE	POSITIVE
558	MANABÍ	LARGE	WARM	POSITIVE	NEGATIVE	NEGATIVE	NEGATIVE	POSITIVE	POSITIVE
559	MANABÍ	MEDIUM	WARM	POSITIVE	NEGATIVE	POSITIVE	NEGATIVE	NEGATIVE	POSITIVE
560	MANABÍ	MEDIUM	WARM	POSITIVE	POSITIVE	POSITIVE	POSITIVE	NEGATIVE	POSITIVE
561	MANABÍ	MEDIUM	WARM	POSITIVE	NEGATIVE	POSITIVE	NEGATIVE	NEGATIVE	POSITIVE
562	MANABÍ	LARGE	WARM	NEGATIVE	NEGATIVE	NEGATIVE	NEGATIVE	NEGATIVE	NEGATIVE
563	MANABÍ	SMALL	WARM	POSITIVE	POSITIVE	POSITIVE	POSITIVE	NEGATIVE	POSITIVE
564	MANABÍ	MEDIUM	WARM	POSITIVE	NEGATIVE	NEGATIVE	NEGATIVE	POSITIVE	POSITIVE
565	MANABÍ	MEDIUM	WARM	POSITIVE	NEGATIVE	NEGATIVE	NEGATIVE	POSITIVE	POSITIVE
566	MANABÍ	SMALL	WARM	POSITIVE	NEGATIVE	POSITIVE	NEGATIVE	NEGATIVE	POSITIVE
567	MANABÍ	MEDIUM	WARM	POSITIVE	NEGATIVE	NEGATIVE	NEGATIVE	POSITIVE	POSITIVE
568	MANABÍ	LARGE	WARM	POSITIVE	NEGATIVE	NEGATIVE	NEGATIVE	POSITIVE	POSITIVE
569	MANABÍ	SMALL	WARM	POSITIVE	NEGATIVE	NEGATIVE	NEGATIVE	POSITIVE	POSITIVE
570	MANABÍ	MEDIUM	WARM	NEGATIVE	NEGATIVE	NEGATIVE	NEGATIVE	NEGATIVE	NEGATIVE
571	MANABÍ	MEDIUM	WARM	POSITIVE	NEGATIVE	NEGATIVE	NEGATIVE	POSITIVE	POSITIVE
572	MANABÍ	LARGE	WARM	POSITIVE	NEGATIVE	POSITIVE	NEGATIVE	NEGATIVE	POSITIVE
573	MANABÍ	SMALL	WARM	POSITIVE	NEGATIVE	POSITIVE	NEGATIVE	NEGATIVE	POSITIVE
574	MANABÍ	MEDIUM	WARM	NEGATIVE	NEGATIVE	NEGATIVE	NEGATIVE	NEGATIVE	NEGATIVE
575	MANABÍ	LARGE	WARM	POSITIVE	NEGATIVE	NEGATIVE	NEGATIVE	POSITIVE	POSITIVE
576	PICHINCHA	SMALL	WARM	POSITIVE	NEGATIVE	NEGATIVE	NEGATIVE	POSITIVE	POSITIVE
577	PICHINCHA	MEDIUM	WARM	POSITIVE	NEGATIVE	NEGATIVE	NEGATIVE	POSITIVE	POSITIVE
578	PICHINCHA	LARGE	WARM	POSITIVE	NEGATIVE	POSITIVE	NEGATIVE	NEGATIVE	POSITIVE
579	PICHINCHA	SMALL	WARM	POSITIVE	NEGATIVE	POSITIVE	NEGATIVE	NEGATIVE	POSITIVE
580	PICHINCHA	MEDIUM	WARM	NEGATIVE	NEGATIVE	NEGATIVE	NEGATIVE	NEGATIVE	NEGATIVE
581	PICHINCHA	MEDIUM	WARM	NEGATIVE	NEGATIVE	NEGATIVE	NEGATIVE	NEGATIVE	NEGATIVE
582	PICHINCHA	SMALL	WARM	NEGATIVE	NEGATIVE	NEGATIVE	NEGATIVE	NEGATIVE	NEGATIVE

583	PICHINCHA	MEDIUM	WARM	POSITIVE	NEGATIVE	POSITIVE	NEGATIVE	NEGATIVE	POSITIVE
584	PICHINCHA	SMALL	WARM	POSITIVE	NEGATIVE	NEGATIVE	NEGATIVE	POSITIVE	POSITIVE
585	PICHINCHA	LARGE	WARM	NEGATIVE	NEGATIVE	NEGATIVE	NEGATIVE	NEGATIVE	NEGATIVE
586	PICHINCHA	LARGE	WARM	NEGATIVE	NEGATIVE	NEGATIVE	NEGATIVE	NEGATIVE	NEGATIVE
587	PICHINCHA	SMALL	WARM	NEGATIVE	NEGATIVE	NEGATIVE	NEGATIVE	NEGATIVE	NEGATIVE
588	PICHINCHA	SMALL	WARM	NEGATIVE	NEGATIVE	NEGATIVE	NEGATIVE	NEGATIVE	NEGATIVE
589	PICHINCHA	SMALL	WARM	NEGATIVE	NEGATIVE	NEGATIVE	NEGATIVE	NEGATIVE	NEGATIVE
590	PICHINCHA	LARGE	WARM	POSITIVE	NEGATIVE	POSITIVE	NEGATIVE	NEGATIVE	NEGATIVE
591	PICHINCHA	SMALL	WARM	NEGATIVE	NEGATIVE	NEGATIVE	NEGATIVE	NEGATIVE	NEGATIVE
592	PICHINCHA	MEDIUM	WARM	POSITIVE	NEGATIVE	POSITIVE	NEGATIVE	NEGATIVE	POSITIVE
593	PICHINCHA	SMALL	WARM	NEGATIVE	NEGATIVE	NEGATIVE	NEGATIVE	NEGATIVE	NEGATIVE
594	PICHINCHA	LARGE	WARM	NEGATIVE	NEGATIVE	NEGATIVE	NEGATIVE	NEGATIVE	NEGATIVE
595	PICHINCHA	MEDIUM	WARM	NEGATIVE	NEGATIVE	NEGATIVE	NEGATIVE	NEGATIVE	NEGATIVE
596	PICHINCHA	SMALL	WARM	POSITIVE	NEGATIVE	POSITIVE	NEGATIVE	NEGATIVE	POSITIVE
597	PICHINCHA	LARGE	WARM	NEGATIVE	NEGATIVE	NEGATIVE	NEGATIVE	NEGATIVE	NEGATIVE
598	PICHINCHA	SMALL	WARM	POSITIVE	NEGATIVE	POSITIVE	NEGATIVE	NEGATIVE	NEGATIVE
599	PICHINCHA	SMALL	WARM	NEGATIVE	NEGATIVE	NEGATIVE	NEGATIVE	NEGATIVE	NEGATIVE
600	PICHINCHA	MEDIUM	WARM	NEGATIVE	NEGATIVE	NEGATIVE	NEGATIVE	NEGATIVE	NEGATIVE
601	PICHINCHA	MEDIUM	WARM	NEGATIVE	NEGATIVE	NEGATIVE	NEGATIVE	NEGATIVE	NEGATIVE
602	PICHINCHA	SMALL	WARM	NEGATIVE	NEGATIVE	NEGATIVE	NEGATIVE	NEGATIVE	NEGATIVE
603	PICHINCHA	LARGE	WARM	NEGATIVE	NEGATIVE	NEGATIVE	NEGATIVE	NEGATIVE	NEGATIVE
604	PICHINCHA	LARGE	WARM	POSITIVE	POSITIVE	POSITIVE	POSITIVE	NEGATIVE	POSITIVE
605	PICHINCHA	MEDIUM	WARM	NEGATIVE	NEGATIVE	NEGATIVE	NEGATIVE	NEGATIVE	NEGATIVE
606	PICHINCHA	MEDIUM	WARM	POSITIVE	NEGATIVE	NEGATIVE	NEGATIVE	POSITIVE	POSITIVE
607	PICHINCHA	LARGE	WARM	NEGATIVE	NEGATIVE	NEGATIVE	NEGATIVE	NEGATIVE	NEGATIVE
608	PICHINCHA	MEDIUM	WARM	POSITIVE	POSITIVE	NEGATIVE	NEGATIVE	NEGATIVE	NEGATIVE
609	PICHINCHA	SMALL	WARM	POSITIVE	POSITIVE	POSITIVE	POSITIVE	NEGATIVE	POSITIVE
610	PICHINCHA	MEDIUM	WARM	POSITIVE	NEGATIVE	POSITIVE	NEGATIVE	NEGATIVE	POSITIVE
611	PICHINCHA	SMALL	WARM	NEGATIVE	NEGATIVE	NEGATIVE	NEGATIVE	NEGATIVE	NEGATIVE
612	PICHINCHA	SMALL	WARM	NEGATIVE	NEGATIVE	NEGATIVE	NEGATIVE	NEGATIVE	NEGATIVE
613	PICHINCHA	LARGE	WARM	NEGATIVE	NEGATIVE	NEGATIVE	NEGATIVE	NEGATIVE	NEGATIVE

614	PICHINCHA	SMALL	WARM	POSITIVE	POSITIVE	POSITIVE	POSITIVE	NEGATIVE	POSITIVE
615	PICHINCHA	MEDIUM	WARM	POSITIVE	NEGATIVE	NEGATIVE	NEGATIVE	POSITIVE	POSITIVE
616	PICHINCHA	LARGE	WARM	NEGATIVE	NEGATIVE	NEGATIVE	NEGATIVE	NEGATIVE	NEGATIVE
617	PICHINCHA	SMALL	WARM	NEGATIVE	NEGATIVE	NEGATIVE	NEGATIVE	NEGATIVE	NEGATIVE
618	PICHINCHA	SMALL	WARM	NEGATIVE	NEGATIVE	NEGATIVE	NEGATIVE	NEGATIVE	NEGATIVE
619	PICHINCHA	SMALL	WARM	POSITIVE	POSITIVE	NEGATIVE	NEGATIVE	NEGATIVE	NEGATIVE
620	PICHINCHA	SMALL	WARM	POSITIVE	NEGATIVE	NEGATIVE	NEGATIVE	POSITIVE	POSITIVE
621	PICHINCHA	SMALL	WARM	POSITIVE	NEGATIVE	POSITIVE	NEGATIVE	NEGATIVE	POSITIVE
622	PICHINCHA	MEDIUM	WARM	POSITIVE	POSITIVE	NEGATIVE	NEGATIVE	NEGATIVE	NEGATIVE
623	PICHINCHA	MEDIUM	WARM	POSITIVE	NEGATIVE	POSITIVE	NEGATIVE	NEGATIVE	POSITIVE
624	PICHINCHA	MEDIUM	WARM	NEGATIVE	NEGATIVE	NEGATIVE	NEGATIVE	NEGATIVE	NEGATIVE
625	PICHINCHA	LARGE	WARM	NEGATIVE	NEGATIVE	NEGATIVE	NEGATIVE	NEGATIVE	NEGATIVE
626	PICHINCHA	LARGE	WARM	POSITIVE	NEGATIVE	NEGATIVE	NEGATIVE	POSITIVE	POSITIVE
627	PICHINCHA	MEDIUM	WARM	POSITIVE	NEGATIVE	NEGATIVE	NEGATIVE	POSITIVE	NEGATIVE
628	PICHINCHA	SMALL	WARM	POSITIVE	NEGATIVE	POSITIVE	NEGATIVE	NEGATIVE	POSITIVE
629	PICHINCHA	MEDIUM	WARM	POSITIVE	POSITIVE	NEGATIVE	NEGATIVE	NEGATIVE	POSITIVE
630	PICHINCHA	LARGE	WARM	POSITIVE	NEGATIVE	NEGATIVE	NEGATIVE	POSITIVE	POSITIVE
631	PICHINCHA	LARGE	WARM	POSITIVE	POSITIVE	NEGATIVE	NEGATIVE	NEGATIVE	POSITIVE
632	PICHINCHA	SMALL	WARM	POSITIVE	POSITIVE	POSITIVE	POSITIVE	NEGATIVE	POSITIVE
633	PICHINCHA	SMALL	WARM	POSITIVE	POSITIVE	POSITIVE	POSITIVE	NEGATIVE	POSITIVE
